# Assembly processes underlying bacterial community differentiation among geographically close mangrove forests

**DOI:** 10.1002/mlf2.12060

**Published:** 2023-03-23

**Authors:** Lu Liu, Nan Wang, Min Liu, Zixiao Guo, Suhua Shi

**Affiliations:** ^1^ State Key Laboratory of Biocontrol, Guangdong Key Lab of Plant Resources, Southern Marine Science and Engineering Guangdong Laboratory (Zhuhai), School of Life Sciences Sun Yat‐Sen University Guangzhou China

**Keywords:** assembly, bacterial community, dispersal limitation, intertidal zone, mangroves

## Abstract

Bacterial communities play pivotal roles in nutrient cycling in mangrove forests. The assembly of mangrove microbial communities has been found to be influenced by complex factors, such as geographic distance, physicochemical conditions, and plant identity, but the relative importance of these factors and how these factors shape the assembling process remain elusive. We analyzed the bacterial communities sampled from three mangrove species (*Aegiceras corniculatum*, *Bruguiera sexangula*, and *Kandelia obovata*) at three locations along the estuarine Dongzhai Harbor in Hainan, China. We revealed larger differences in rhizosphere bacterial communities among geographical locations than among plant species, indicated by differences in diversity, composition, and interaction networks. We found that dispersal limitation and homogeneous selection have substantial contributions to the assembly of mangrove rhizosphere bacterial communities in all three locations. Following the phylogenetic‐bin‐based null model analysis (iCAMP) framework, we also found dispersal limitation and homogeneous selection showing dominance in some bins. The greater differences among geographic locations may be mainly attributed to the larger proportions of dispersal limitation even at such a short geographic distance. We also found that beta diversity was positively correlated with environmental distances, implying that the more similar environmental conditions (such as rich carbon and nitrogen contents) among plant species may have shaped similar bacterial communities. We concluded that the geographic distances, which are associated with dispersal limitation, played a key role in assembling mangrove rhizosphere bacterial communities, while physicochemical conditions and plant identity contributed less.

## Impact statement

Mangrove forests are ecologically essential for protecting coasts, supporting coastal biodiversity, and carbon sequestration. Bacterial communities are pivotal for the growth and health of mangrove plants. The conservation and restoration of mangrove forests necessitate a substantial understanding of bacterial community assembly in mangrove ecosystems. Our study found greater differentiation among geographic locations than among plant species. This pattern is attributed to the key role of dispersal limitation in assembling the rhizosphere bacterial communities of mangrove plants. Given that the three locations along the estuary that we studied are very close, such differentiation due to dispersal limitation could be general among different mangrove forests that are usually distributed at larger geographic scales. These findings deepen our knowledge of mangrove bacterial communities.

## INTRODUCTION

Mangrove forests feature woody plants inundated by salty seawater and are found in tropical and subtropical intertidal zones. Although there are only ~70 mangrove species worldwide, mangroves are considered the most critical tropical ecosystems due to their functions in protecting coasts, mitigating typhoons, and carbon sequestration^1^. Mangrove ecosystems are among the most productive ecosystems in the world due to the high turnover rates of nutrient cycling^2^, and they have supported high animal diversity. Microbial communities played essential roles in nutrient cycling. As the intertidal environments highly fluctuate, it is interesting to investigate how the microbial communities in the mangrove ecosystems are assembled.

Microbial communities are shaped by a combination of deterministic and stochastic processes. The deterministic mechanisms assume that community assembly results from the predictable filtering of species by both physicochemical and biotic conditions^3^. Various physicochemical factors have an influence on the assembly process, resulting in differences among rhizosphere microbial communities. The deterministic processes have been found to better explain the prokaryotic communities of mangroves in Southeastern China^4^, ^5^. The mean annual precipitation and total organic carbon were important factors in explaining the variations of microbial communities of mangrove sediments in Southeastern China^4^. Recently, the bacterial community composition was shown to vary more strongly along an intertidal gradient within each mangrove forest than between forests in different geographic regions, indicating a strong effect of local environmental factors^6^.

Interestingly, significant differences among microbial communities associated with different mangrove species within the same mangrove forest have been repeatedly reported^7^, ^8^, ^9^, ^10^, ^11^. The plant–microbe interactions, in which plant root exudates provide nutrients for microbes while microbes transform nutrients into carbon, nitrogen, and other elements for plants, likely play a role in shaping microbial communities^2^, ^12^.

On the other hand, the neutral community model (NCM) assumes that random birth, death, dispersal, and drift events greatly influence the structure of microbial communities^13^. A previous study has argued that geographic scales, in addition to physicochemical gradients, determined microbial community assembly^14^. Geographic distance was found to influence the assembly of fungal communities of mangroves in different locations in Southeastern China, mainly due to the dispersal limitation among locations^15^. The relative contribution of deterministic and stochastic (neutral) factors in assembling mangrove rhizosphere microbial communities remains elusive.

Mangrove forests located in estuarine regions provide a special chance to study the influence of complex conditions on microbial community assembly. Estuaries have a physicochemical gradient from freshwater to seawater and inundation gradients from occasional to prolonged. Mangrove growth and development necessitate an appropriate salinity^16^, and the growth of mangroves changes the carbon and nitrogen content in sediments. Mangrove plants along an estuary naturally inhabit a gradient of physicochemical conditions from downstream regions to upstream regions.

Hence, we used high‐throughput 16S rRNA gene sequencing to capture the bacterial communities of rhizosphere sediment of *Aegiceras corniculatum*, *Bruguiera sexangula*, and *Kandelia obovata*, as well as bulk sediments without plant roots. Parallel samples were collected from three locations: upstream, midstream, and downstream of the Dongzhai Harbor on Hainan Island, China. By analyzing the bacterial communities sampled from rhizospheres of different mangrove species along an estuary, we aimed to test two hypotheses: (1) the rhizosphere bacterial communities would show larger differences among geographic locations along the estuary than among different plant species and (2) the differences among geographic locations can be mostly attributed to dispersal limitation.

## RESULTS

### Diversity of bacterial communities in different locations and different plant rhizospheres

**Figure 1:**
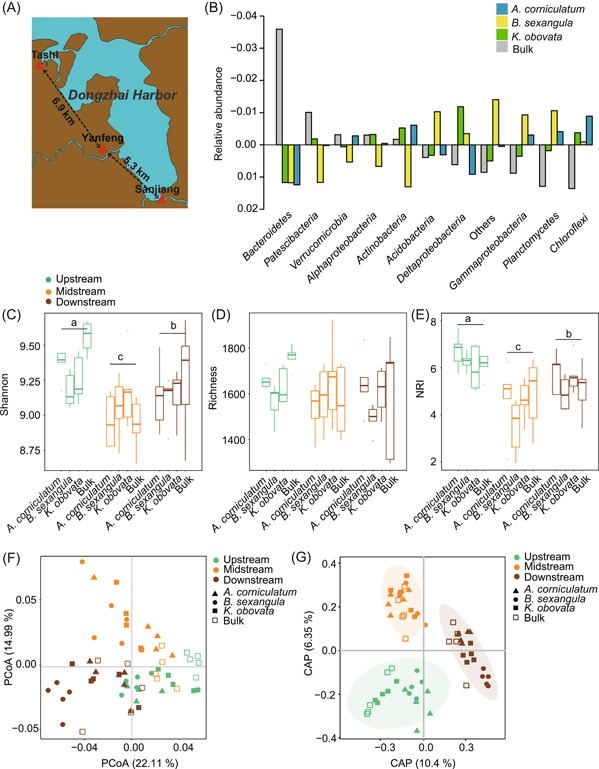
Bacterial community diversity and structure of three mangrove forests along Dongzhai Harbor in Hainan, China. (A) The map shows the sampled geographical locations in the Dongzhai National Mangrove Nature Reserve. The map was modified from a base map from Natural Earth (https://www.naturalearthdata.com/). (B) Variation of the bacterial community composition of the three mangroves and the bulk. In each taxonomic group, the bars show the deviation to the group mean. (C–E) Shannon index, richness index, and Net Relatedness index (NRI) of different samples. The lowercase letters indicate significant differences (*p* < 0.05). (F) PCoA plot of the weighted UniFrac distances between samples. (G) Canonical analysis of principal coordinates (CAP) plot of the Bray–Curtis distances between all samples. The first two CAPs explained 16.75% of the variance (*p* < 0.001).

We sampled rhizosphere sediments from individual trees of three mangrove species (*A. corniculatum*, *B. sexangula*, and *K. obovata*) at Sanjiang (upstream), Yanfeng (midstream), and Tashi (Downstream) in Dongzhai Harbor, Hainan, China (Figure 1A). In each of the three locations, we collected five rhizosphere sediments for each plant species and five bulk sediments, resulting in a total of 60 sediment samples. A total of 1,281,802 sequences of 16S rRNA were obtained across the 60 sediment samples. Overall, 7135 operational taxonomic units (OTUs) were annotated at 97% identity. *Proteobacteria* (including *Alphaproteobacteria*, *Deltaproteobacteria*, and *Gammaproteobacteria*) was the dominant phylum, with an average relative abundance accounting for 39% of the total amplicons, followed by *Bacteroidetes*, *Planctomycetes*, *Chloroflexi*, and *Acidobacteria* (Table S1). The most dominant orders are *Desulfobacterales*, *Bacteroidales*, *Anaerolineales*, *Steroidobacterales*, and *Flavobacteriales* (Figure S1A). The abundances differed obviously among the four sediment types (Figure 1B). The relative abundances of *Desulfobacterales*, *Bacteroidales*, and *Ignavibacteriales* were much higher in the rhizosphere than in bulk sediments, while *Anaerolineales* was more abundant in bulk communities (Figure S1A). At the family level, *Desulfobulbaceae*, *Prolixibacteraceae*, *Bacteroidetes* BD2‐2, *Kiritimatiellaceae*, and *Ignavibacteriaceae* were more abundant in the rhizosphere than in the bulk sediments (Figure S1B).

The Tukey–HSD (honest significant differences) test indicated significant differences in the Shannon index of the bacterial communities at upstream, midstream, and downstream regions (*p* < 0.05, Figure 1C), but no significant difference in the richness index (Figure 1D). Within each location, the alpha diversity (indicated by Shannon and richness indexes) showed no significant difference among rhizospheres of different plant species or between rhizospheres and bulk sediments (*p* > 0.05, Figure 1C,D). In addition, most samples have Net Relatedness Index (NRI) values > 1.96, suggesting that the bacterial communities are phylogenetically clustered (Figure 1E). The midstream region has the lowest NRI values, while the upstream region has the highest NRI values (Figure 1E).

We determined the beta diversity of bacterial communities among geographical locations and plant species. The unconstrained principal coordinate analysis (PCoA) indicated that the bacterial communities were differentiated mainly by geographical locations (Figure 1F). We also used permutational multivariate analysis of variance (PERMANOVA) to test the clustering by plant species, by geographic locations, or by both plant species and geographic locations, and found that the most significant grouping was by geographic locations (Bray–Curtis distance *R* ^2^ = 0.256 and Weighted UniFrac distance *R* ^2^ = 0.307, *p* < 0.001, Table S2). Furthermore, the canonical analysis of principal coordinates (CAP) showed clear clustering of the samples into three groups by geographical locations (Figure 1G). The first two principal coordinates explained 16.75% of the total variance (*p* < 0.001).

We used both PERMANOVA and analysis of similarities (ANOSIM) to test the significance of the difference in each pair of geographical locations (Table S3) and each pair of sediment types (Table S4). We found significant differences in every pair of geographic locations, regardless of the identity of plant species (Bray–Curtis distance: ANOSIM *R* = 0.44–1.00, PERMANOVA *R* ^2^ = 0.23–0.55, *p* < 0.05; Weighted UniFrac distance: ANOSIM *R* = 0.30–0.95, PERMANOVA *R* ^2^ = 0.25–0.73, *p* < 0.05; Table S3). In contrast, much lower levels of difference were found between rhizosphere sediments of different plant species (Bray–Curtis distance: ANOSIM *R* = 0.06–0.17, PERMANOVA *R* ^2^ = 0.05–0.10; Weighted UniFrac distance: ANOSIM *R* = 0.02–0.14, PERMANOVA *R* ^2^ = 0.03–0.13; Table S4), and the rhizosphere bacterial communities of *A. corniculatum* and *K. obovata* were not significantly different (Table S4).

### Differences in the composition of bacterial communities

**Figure 2:**
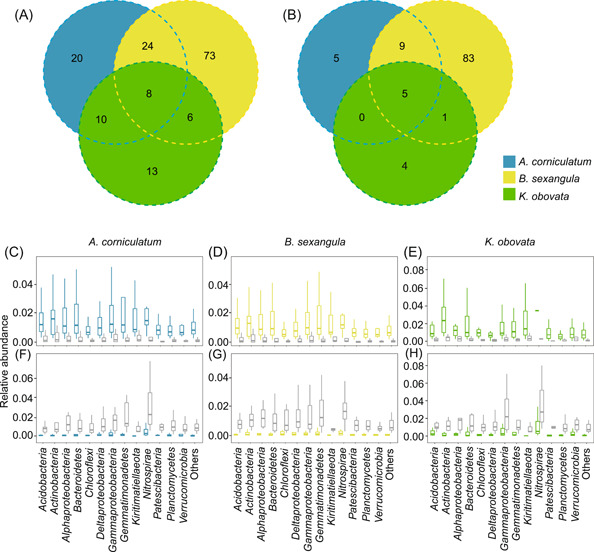
Taxonomic comparisons of the bacterial communities of rhizosphere and bulk samples. (A) Numbers of the enriched operational taxonomic units (OTUs) in rhizosphere samples compared to bulk samples. (B) Numbers of the depleted OTUs in rhizosphere samples compared to bulk samples. (C–E) Abundance distributions of the OTUs that are enriched in mangrove rhizosphere microbial communities (colored boxes), relative to the bulk microbial communities (gray boxes). (F–H) Abundance distributions of the OTUs that are depleted in mangrove rhizosphere microbial communities (colored boxes), relative to the bulk microbial communities (gray boxes). In (C–H), the OTUs belonging to the same taxonomic group are plotted in the same box. For each OTU, there are three abundance values of rhizosphere samples (average values of upstream, midstream, and downstream samples) and three values of bulk samples (upstream, midstream, and downstream).

To identify what OTUs have been enriched or depleted in the rhizosphere bacterial communities of the three mangrove species relative to the bulk sediments, we conducted differential abundance analyses on the negative binomial distributions of OTU counts. For the rhizosphere samples of each plant species, if an OTU was enriched (or depleted) at no less than two of the three geographic locations, it was considered enriched (or depleted) in this plant species. This analysis was conducted on the top 1493 OTUs with high abundance, which accumulated to 75.5% of the total abundance of the rhizosphere bacterial community and 63.08% of the total abundance of the bulk bacterial community. Among these OTUs, 62, 111, and 37 were identified as being enriched in the bacterial communities of *A. corniculatum*, *B. sexangula*, and *K. obovata*, respectively (Figure 2A), while 19, 98, and 10 were identified as being depleted (Figure 2B). In total, 154 OTUs were enriched, while 107 were depleted in at least one of the three mangrove species (Figure 2A,B). *Actinobacteria*, *Alphaproteobacteria*, *Bacteroidetes*, and *Kiritimatiellaeota* were enriched with higher frequency in the rhizosphere sediments than in the bulk sediments (Figure 2C–E), while *Nitrospirae* was largely depleted in the rhizosphere sediments (Figure 2F–H).

### Bacterial networks differed more among geographic locations than among plant species

We constructed networks for rhizosphere samples by geographical locations (Table S5). The *R* ^2^ of the power law ranged from 0.818 to 0.963 in the three networks, indicating that the distributions of the degree centrality were scale‐free (Table S5), that is, the networks were not constructed randomly. Notably, the statistics of the harmonic geodesic distance (HD), the average clustering coefficient (avgCC), and modularity were generally larger than those of the networks simulated randomly (Table S5), indicating that the observed interactions among nodes are biologically significant. In each network, nodes (one node for one OTU) were identified if the abundance of a pair of OTUs varied among samples with significant correlation.

**Figure 3:**
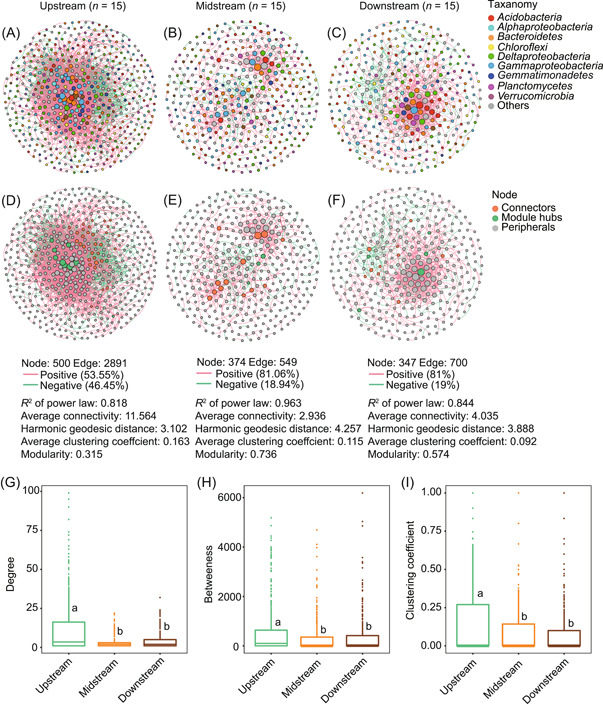
Features of the rhizosphere bacterial community networks across the three geographic locations. Overview of the rhizosphere bacterial networks across the three locations; each location contained 15 rhizosphere samples. Node size is proportional to node connectivity, and colors indicate different taxonomies (A–C) or node properties (D–F). (G–I) Degree, betweenness, and clustering coefficient of each node in the networks of the three geographic locations. Different lowercase letters indicate significantly different groups.

We found strong differences among the networks of the three geographic locations. The node numbers ranged from 347 to 500, while the number of edges decreased drastically from 2891 in the upstream region to 700 in the downstream region and only 549 in the midstream region. The values of average connectivity also differed remarkably (11.564 vs. 2.936 and 4.035) (Figure 3A–F). Negative interaction is relatively high in the upstream region, but positive interaction is dominant in the other two locations (Figure 3A–F). Meanwhile, the upstream network is less modulated than the networks of midstream and downstream regions (Figure 3A–F). Consistently, the upstream network shows larger values of degree, betweenness, and clustering coefficient than the midstream and downstream networks (Figure 3G–I). Hence, the microbial network of the upstream mangroves is denser and more connected but less modulated than those of the midstream and downstream mangroves.

The nodes of all three networks were overrepresented by taxonomic groups of *Deltaproteobacteria* (12.83%–17.00% of all nodes), *Gammaproteobacteria* (15.51%–17.29%), *Bacteroidetes* (11.80%–12.68%), and *Planctomycetes* (8.07%–10.70%) (Table S6). In particular, 85 OTUs were common among all three networks.

We also constructed interaction networks for the three plant species. We observed an increasing number of nodes and edges from *A. corniculatum*, *B. sexangula* to *K. obovata* (Figure S2A–C). *A. corniculatum* has a relatively lower degree, average connectivity, and clustering coefficient, but larger HD and modularity than the other two plants (Figure S2). In all three plant species, there are more positive interactions than negative interactions (Figure S2A–C). Overall, the differences among the three plant species are less than those among the three geographic locations.

### Assembly mechanisms of rhizosphere bacterial communities of mangroves

The relative contributions of different mechanisms in shaping the bacterial communities of mangroves were quantified using three methods: the NCM, habitat niche breadths (community‐level *B* value [*Bcom*]), and phylogenetic‐bin‐based null model analysis (iCAMP). NCM assumes that the frequency of the occurrence of OTUs is correlated with the mean relative abundance. *R* ^2^ evaluates how the observed pattern fits the NCM. The *R* ^2^ value calculated by combining all samples is 0.801 (Figure S3A). For each of *A. corniculatum, B. sexangula, K. obovata*, and the bulk, the values were 0.729, 0.704, 0.700, and 0.679, respectively (Figure S3B–E). These values indicated that 72.9%, 70.4%, 70.0%, and 67.9% of the OTUs were within the 95% confidential intervals of the NCM expectation. Correspondingly, the migration rates (*m* values) were 0.814, 0.706, 0.727, and 0.566, respectively, suggesting a higher level of dispersal in the rhizosphere than in the bulk bacterial communities. For the rhizosphere bacterial communities at the upstream, midstream, and downstream regions, the *R* ^2^ values were 0.710, 0.760, and 0.718, corresponding to migration rates of 0.725, 0.977, and 0.936, respectively (Figure S3F–H). Consistently, we also observed significantly larger *Bcom* values in the rhizosphere of the three plants than in bulk sediments (Figure S4A), as well as significantly larger *Bcom* values at the midstream region than at the other two regions (Figure S4B). Interestingly, the *Bcom* values showed a significant positive correlation with carbon content, nitrogen content, and salinity, but a significant negative correlation with pH (Figure S4C–F). Notably, the computation of *Bcom* values does not take environmental conditions into consideration; thus, the comparisons of *Bcom* values should be interpreted with caution.

**Figure 4:**
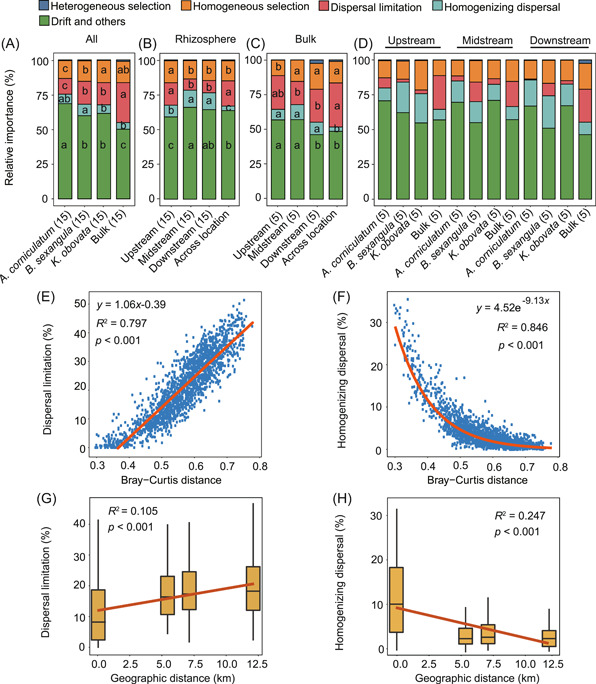
Mechanisms of bacterial community assembly in mangroves. (A–D) Proportions of different assembly mechanisms that were computed for the four sediment types (A), rhizosphere sediments of the three geographic locations (B), bulk sediments of the three geographic locations (C), and each sediment type at each location (D). Notably, “across location” indicates that the computation was conducted by pooling the samples from all three locations. Different lowercase letters indicate significantly different groups. Significance was determined from the Duncan test of one‐way analysis of variance, with a cutoff < 0.05. (E, F) Correlations of the relative importance of dispersal limitation and homogenizing dispersal with Bray–Curtis distances. (G, H) Correlations of the relative importance of dispersal limitation and homogenizing dispersal with geographic distances.

Using the iCAMP^17^ analysis, the proportional contributions of dispersal limitation, homogenizing dispersal, heterogeneous selection, homogeneous selection, and “drift and others” were estimated. In both analyses conducted by sediment type and by geographic locations, the dispersal limitation and homogeneous selection have shown substantial contributions but the heterogeneous selection and homogenizing dispersal have contributed much less (Figure 4A–C, Table S7). Notably, “drift and others” usually has the largest proportion (Figure 4A–C, Table S7). When we applied the computation to individual sediment types at each location, we found that the rhizosphere samples have lower levels of dispersal limitation but higher levels of homogenizing dispersal, in comparison to the bulk samples (Figure 4D).

Interestingly, we found a positive correlation between proportions of dispersal limitation and Bray–Curtis distances (Figure 4E) and strong negative correlations between proportions of homogenizing dispersal and Bray–Curtis distances (Figure 4F). A similar pattern was also observed between these two assembly mechanisms and geographic distance (Figure 4G,H). However, much weaker though significant correlations were observed between proportions of homogeneous selection (or heterogeneous selection) and Bray–Curtis distances (Figure S5). This implied that dispersal limitation is the major mechanism for increasing the beta diversity of mangrove bacterial communities while homogenizing dispersal is the mechanism for decreasing beta diversity.

We also used the null model of stegen^18^ to estimate the relative contribution of these mechanisms, which revealed smaller proportions of “drift and others” in almost all geographic locations or plant species (Figure S6). However, this estimation also showed that dispersal limitation has played a key role (Figure S6). Moreover, the correlations between the proportions of these mechanisms with Bray–Curtis distances or with geographic distances showed patterns that were consistent with those revealed by the iCAMP analysis described above (Figure S6).

### Variation of assembly mechanisms across different phylogenetic groups

**Figure 5:**
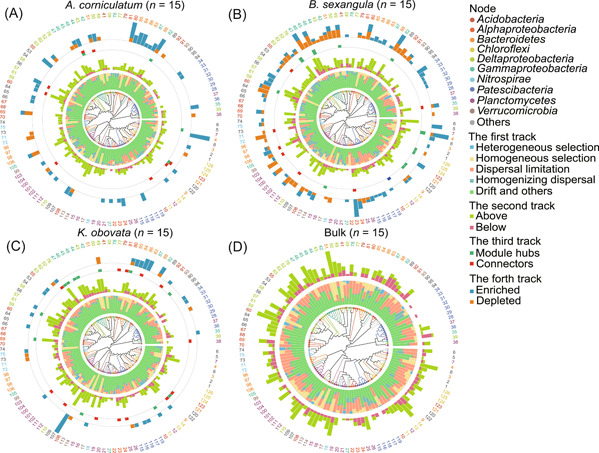
Variation of assembly mechanisms across different phylogenetic bins. (A–C) Plot of *Aegiceras corniculatum* (A), *Bruguiera sexangula* (B), and *Kandelia obovata* (C). At the center is a phylogenetic tree. From inner to outer, the four tracks present the relative importance of different assembly mechanisms in each bin (the first track), the number of non‐neutral community model OTUs (the second track), the number of network‐keystone OTUs (the third track), and the number of enriched/depleted OTUs (the fourth track). (D) Plot of the bulk samples, with the proportions of different assembly mechanisms in each bin presented.

We followed the iCAMP framework to quantify the relative importance of different mechanisms (homogeneous selection, heterogeneous selection, dispersal limitation, homogenizing dispersal, and “drift and others”) at the level of individual phylogenetic lineages (bins). We applied the method to rhizosphere samples of each mangrove species and the bulk. The 7135 OTUs were divided into 126 phylogenetic bins. Most of the bins (109, 101, 104, and 82 bins for *A. corniculatum*, *B. sexangula*, *K. obovata*, and the bulk, respectively) showed large proportions of “drift and others” (74.33%, 69.70%, 72.68%, and 63.44% at average), indicating that the assembly of most bins is largely unresolved (Figures 5 and S7). Nonetheless, dispersal limitation is dominant in 8, 14, 8, and 34 bins in these samples (Figures 5 and S7, Table S8). Among these bins, nine bins belong to the phylum *Patescibacteria* and seven bins belong to the phylum *Acidobacteria*. Bin115 (Order *ABY1*, Phylum *Patescibacteria*) and Bin71 (Order *Thermodesulfovibrionia*, Phylum *Nitrospirae*) were dominated by dispersal limitation in all four sediments, while Bin83 (Genus *Hoppeia*, Phylum *Bacteroidetes*) was dominated in the three rhizosphere sediments. However, only Bin37 (Phylum *Planctomycetes*) was dominated by homogenizing dispersal in the rhizosphere sediment of *B. sexangula*.

Homogeneous selection is also dominant in 9, 10, 14, and 9 bins of *A. corniculatum*, *B. sexangula*, *K. obovata*, and the bulk, respectively (Figures 5 and S7, Table S8), but heterogeneous selection is not dominant in any of the bins. Notably, Bin114 (Family *Pedosphaeraceae*, Phylum *Verrucomicrobia*), Bin17 (Family *Rubritaleaceae*, Phylum *Verrucomicrobia*), and Bin59 (Family *Desulfobacteraceae*, Class *Deltaproteobacteria*) were dominated by homogeneous selection in rhizospheres of all three mangroves as well as in the bulk sediments. Bin34 (Order *Parcubacteria*, Phylum *Patescibacteria*), Bin75 (Order *Thermodesulfovibrionia*, Phylum *Nitrospirae*), Bin 80 (Family *Sphingomonadace*, Class *Alphaproteobacteria*), and Bin92 (Phylum *Acidobacteria*) were dominated by homogeneous selection in rhizospheres of the three mangroves but not in the bulk sediments. In contrast, Bin119 (Order *ABY1*, Phylum *Patescibacteria*) and Bin85 (Family *Prolixibacteraceae*, Phylum *Bacteroidetes*) were dominated by homogeneous selection only in the bulk sediments.

We aligned the assembly mechanisms with the numbers of OTUs enriched/depleted in the rhizosphere samples relative to the bulk samples (enriched/depleted OTUs), the numbers of OTUs above or below the NCM expectation (non‐NCM OTUs), and the numbers of OTUs identified as module hubs or connectors in the network analyses (Network‐keystone OTUs) (Figure 5). We found that the numbers of enriched/depleted, non‐NCM, and Network‐keystone OTUs varied roughly in consistency across different bins. Interestingly, in all three mangrove species, the bins with larger numbers of enriched/depleted, non‐NCM, and Network‐keystone OTUs usually have larger proportions of resolved assembly mechanisms (we refer to dispersal limitation, homogenizing dispersal, homogeneous selection, and heterogeneous selection). In particular, we found that *Acidobacteria* and *Patescibacteria* were simultaneously overrepresented in bins with high proportions of dispersal limitation (Figure 5).

We further explored whether some iCAMP bins have unexpectedly large numbers of non‐NCM OTUs (Figure S8). We found that several bins have disproportionally large numbers of OTUs above the NCM cutoff, including Bin108 (*Acidobacteria*), Bin51 (*Prolixibacteraceae*), Bin59 (*Desulfobacteraceae*), Bin8 (*Actinomarinales*), and Bin80 (*Sphingomonadaceae*). Interestingly, all these bins are dominated by homogeneous selection in the iCAMP analysis. In particular, the *Sphinogomonadaceae* family (Bin80) has the capacity to utilize a wide variety of carbon sources, and several *Sphinogomonadaceae* bacteria are degraders of recalcitrant (xenobiotic) molecules^19^, ^20^. Similarly, we found that Bin71 (*Nitrospirae*), which has a disproportionally large number of OTUs below the NCM cutoff, was dominated by dispersal limitation.

### Physicochemical conditions have an influence on the mangrove bacterial community assembly

Environmental conditions may have an impact on the assembly of bacterial communities. We investigated how the environmental conditions differed among locations and among mangrove species and how these variations have influenced the bacterial communities. Comparing the 60 sediment samples, we found that salinity, carbon content, and nitrogen content were generally higher in the downstream and midstream regions than in the upstream region (*p* < 0.05, Tables 1 and S9). The downstream region has a generally lower pH value than the upstream region, and the rhizosphere sediments have a generally lower pH value than the bulk sediments (*p* < 0.05, Table 1). The moisture, carbon, hydrogen, and nitrogen contents were generally higher in the rhizosphere than in the bulk sediments (*p* < 0.05, Table 1). The carbon content was relatively low in the upstream region, and the upstream bulk sediments has the lowest carbon and nitrogen contents. The rhizosphere sediments of *B. sexangula* and *K. obovata* showed a similar pattern of carbon content with bulk sediments, while *A. corniculatum* showed a contrasting pattern (Table 1).

**Table 1:** Physicochemical conditions in different samples.

Mean ± standard error	Sample size	pH	Salinity (PSU)	Moisture (%)	Total carbon (%)	Total hydrogen (%)	Total nitrogen (%)	Total sulfur (%)	Carbon/nitrogen
Upstream—*Aegiceras corniculatum*	5	6.44 ± 0.09	2.53 ± 0.11*	110.66 ± 9.89*	4.40 ± 0.33*	1.16 ± 0.05*	0.24 ± 0.01*	0.28 ± 0.04	18.58 ± 0.35*
Midstream—*A. corniculatum*	5	6.68 ± 0.05*	2.64 ± 0.09*	86.92 ± 4.29	4.01 ± 0.12*	1.30 ± 0.01*	0.24 ± 0.00*	0.42 ± 0.05*	16.75 ± 0.63
Downstream—*A. corniculatum*	5	6.33 ± 0.11	2.32 ± 0.14	66.75 ± 3.01	3.06 ± 0.16	0.67 ± 0.03	0.18 ± 0.01	0.46 ± 0.03*	17.17 ± 0.27
All—*A. corniculatum*		6.48 ± 0.06bc	2.50 ± 0.07a	88.11 ± 5.91b	3.82 ± 0.19b	0.99 ± 0.07b	0.21 ± 0.01b	0.38 ± 0.03	17.50 ± 0.32b
Upstream—*Bruguiera sexangula*	5	6.28 ± 0.06	2.17 ± 0.16	80.31 ± 2.39	3.57 ± 0.25	1.06 ± 0.04	0.20 ± 0.01	0.27 ± 0.04	17.70 ± 0.29
Midstream—*B. sexangula*	5	6.43 ± 0.08*	2.70 ± 0.21*	97.29 ± 5.70	4.47 ± 0.11	1.30 ± 0.03*	0.24 ± 0.01	0.46 ± 0.10	19.02 ± 0.53*
Downstream—*B. sexangula*	5	6.28 ± 0.03	3.06 ± 0.14*	136.19 ± 7.23*	7.56 ± 0.38*	1.26 ± 0.04*	0.34 ± 0.01*	0.93 ± 0.07*	21.95 ± 0.28*
All—*B. sexangula*		6.33 ± 0.04c	2.64 ± 0.13a	104.60 ± 6.91a	5.20 ± 0.48a	1.21 ± 0.03a	0.26 ± 0.02a	0.55 ± 0.08	19.56 ± 0.52a
Upstream*—Kandelia obovata*	5	6.95 ± 0.04*	2.00 ± 0.11	61.67 ± 2.19	1.20 ± 0.05	0.64 ± 0.03	0.09 ± 0.00	0.11 ± 0.01	13.35 ± 0.43
Midstream—*K. obovata*	5	6.42 ± 0.04	2.62 ± 0.13*	92.47 ± 2.63*	4.81 ± 0.23*	1.31 ± 0.05*	0.25 ± 0.01*	0.68 ± 0.11	19.29 ± 0.73*
Downstream—*K. obovata*	5	6.27 ± 0.13	2.35 ± 0.14*	79.05 ± 4.38*	4.11 ± 0.69*	0.75 ± 0.10	0.20 ± 0.02	0.51 ± 0.06	20.48 ± 1.29*
All*—K. obovata*		6.55 ± 0.09b	2.32 ± 0.10a	77.73 ± 3.78bc	3.37 ± 0.47b	0.90 ± 0.09b	0.18 ± 0.02b	0.43 ± 0.08	17.70 ± 0.96b
Upstream—Bulk	5	6.95 ± 0.07*	1.47 ± 0.17	48.99 ± 3.13	1.08 ± 0.10	0.75 ± 0.10	0.09 ± 0.01	0.08 ± 0.01	11.98 ± 0.36
Midstream—Bulk	5	6.67 ± 0.11	2.42 ± 0.14*	88.26 ± 6.18*	2.98 ± 0.13*	1.14 ± 0.05*	0.18 ± 0.01*	0.73 ± 0.12*	16.35 ± 0.31*
Downstream—Bulk	5	6.73 ± 0.09*	2.05 ± 0.14*	56.27 ± 5.98	2.05 ± 0.14	0.67 ± 0.03	0.12 ± 0.01	0.70 ± 0.07*	16.52 ± 0.70*
All—Bulk		6.78 ± 0.06a	1.98 ± 0.13b	64.51 ± 5.36c	2.04 ± 0.22c	0.85 ± 0.07b	0.13 ± 0.01c	0.51 ± 0.09	14.95 ± 0.62c

* Significantly large values compared with those of the same sediment type in different locations. Different lowercase letters indicate levels of significant differences among sediment types. Significance level was determined by ANOVA (Duncan's multiple range test). ANOVA, analysis of variance; PSU, practical salinity units.

**Figure 6:**
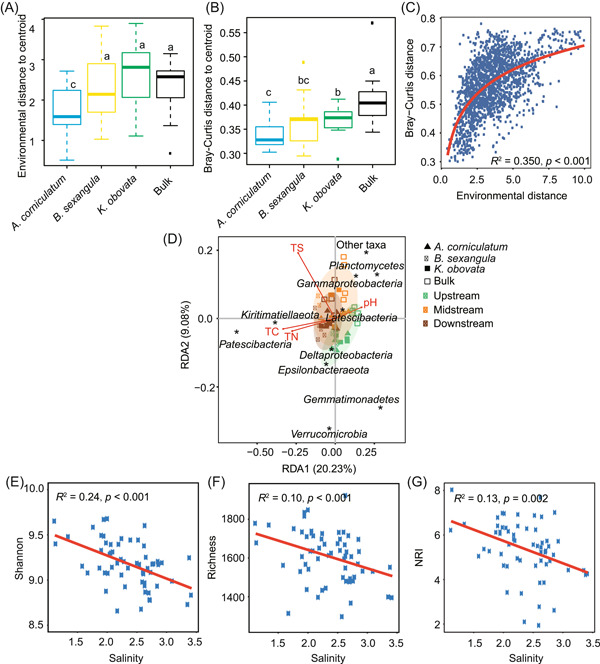
Influence of environmental conditions on the mangrove bacterial communities. (A,B) Environmental distances (A) or Bray–Curtis distances (B) to the centroid in rhizosphere samples of the three mangroves and the bulk samples. The box indicates the range of quartiles, horizontal lines indicate the median, and whiskers indicate the 95% range. (C) Correlation between the environmental distances and Bray–Curtis distances. (D) Correlations between taxonomic groups and physicochemical conditions. Only the physicochemical conditions with a significant correlation (*p* < 0.05) and the top 10 abundant phyla (class for *Proteobacteria*) are labeled in the figure. (E–G) Shannon, richness, and NRI values are negatively correlated with salinity. NRI, Net Relatedness Index; TC, total carbon; TN, total nitrogen; TS, total sulfur.

We computed the environmental distances and Bray–Curtis distances for each plant species, with the bulk sediments as the control. We found that both environmental distances and Bray–Curtis distances increased as *A. corniculatum* < *B. sexangula* < *K. obovata* (Figure 6A,B). The Bray–Curtis dissimilarity was significantly positively correlated with environmental distance (Figure 6C, Table S10). The Bray–Curtis distances were also significantly correlated with geographic distances, and the correlations were even stronger than those between Bray–Curtis distances and environmental distances (Table S10, Figure S9). Moreover, environmental distances themselves were positively correlated with geographic distance (Figure S10). All these results indicated that environmental conditions have a substantial, though weaker than geographical factors, influence on the assembly of mangrove bacterial communities.

We moved on to find the key environmental conditions. Spearman's rank correlation analysis indicated that pH was negatively correlated with carbon (*r*_s_ = −0.59, *p* < 0.001), nitrogen (*r*_s_ = −0.57, *p* < 0.01), the carbon–nitrogen ratio (*r*_s_ = −0.66, *p* < 0.001), and salinity (*r*_s_ = −0.41, *p* < 0.01) in all sediments, while salinity was strongly positively correlated with carbon (*r*_s_ = 0.72, *p* < 0.001) and nitrogen (*r*_s_ = 0.74, *p* < 0.001) contents. Moreover, carbon content was positively correlated with nitrogen, hydrogen, and sulfur contents and the carbon–nitrogen ratio (*r*_s_ ≥ 0.61, *p* < 0.001). In short, salinity, carbon, nitrogen, and the carbon–nitrogen ratio appear to be positively correlated with each other. We performed multiple regression on matrices (MRM analysis) to analyze the key physicochemical factors that shape the bacterial community. In each cluster, if multiple conditions are highly collinear (Spearman's *ρ* ^2^ near 0.5), we use only one of the conditions in the cluster to represent the whole cluster (Figure S11). Hence, salinity, pH, moisture, carbon, sulfur contents, and carbon/nitrogen were retained for the analyses. The MRM analysis showed that the bacterial community structure was significantly influenced by pH, sulfur content, and carbon content (Table S11). We also conducted the same analysis for the rhizosphere microbial community of each mangrove species and the bulk sediment (Table S11). We found that sulfur content and sediment moisture were significant in *A. corniculatum*; sediment moisture was significant in *B. sexangula*; and carbon content and carbon/nitrogen were significant in *K. obovata* (Table S11). In comparison, no environmental factor was significant in bulk bacterial communities (Table S11).

The redundancy analysis (RDA), which was based on the Bray–Curtis distance matrix of the rhizosphere microbial community at the phylum level (class for *Proteobacteria*), showed that pH, total carbon (TC), total nitrogen (TN), and total sulfur (TS) contents were the key physicochemical conditions (Figure 6D). The abundances of *Patescibacteria* and *Kiritimatiellaeota* were strongly positively correlated, and the abundances of *Gammaproteobacteria* and *Planctomycetes* were negatively correlated, with TC and TN contents (Figure 6D). Notably, we found that the Shannon index, richness, and NRI values showed a significantly negative correlation with salinity (Figure 6E–G).

Environmental conditions may have a particular influence on the community assembly mechanisms. We revealed a strong negative correlation between the proportions of dispersal limitation and carbon content (or nitrogen content) (Figure S12), but strong positive correlations between the proportions of homogenizing dispersal and carbon content (or nitrogen content) (Figure S12). Moreover, the two mechanisms show opposite correlations with environmental distances (Figure S12). Consistently, a larger proportion of dispersal limitation and lower homogenizing dispersal were observed in the bulk sediments than in the rhizosphere sediments, likely due to the higher carbon and nitrogen contents in rhizosphere sediments (Table S9). In addition, the proportions of dispersal limitation are positively correlated and the proportions of homogenizing dispersal are negatively correlated with geographic distance (Figures 4G,H and S13). Compared with the values estimated from one location, we indeed observed lager proportions of dispersal limitation but a lower proportion of homogenizing dispersal when all locations were combined in the estimation (Figure 4B,C).

By exploring how the abundance of OTUs in each bin, which was defined in the iCAMP analysis, correlates with environmental conditions, we found that these bins are significantly correlated with different environmental conditions (Figure S14). In particular, Bin25, Bin22, Bin46, and Bin72 are sensitive, and significantly correlated with almost all eight conditions (Figure S14). In contrast, the six bins dominated by homogeneous selection (Bin4, Bin59, Bin51, Bin42, Bin92, and Bin34) are correlated with fewer environmental conditions, implying that these taxa prefer specific conditions (Figure S14).

## DISCUSSION

The influence of spatial and environmental conditions in shaping bacterial communities has been investigated in subtropical bays^21^ and microeukaryotic communities in rivers^22^. By comparing bacterial communities of three mangrove species at three geographic locations along the estuary of Dongzhai Harbor, we found greater differentiation among the three geographic locations than among different mangrove species. This differentiation existed at all levels of diversity, composition, and interaction networks. Although most of the assembly processes of rhizosphere bacterial communities of mangrove species were not resolved (“drift and others”), we found that dispersal limitation and homogeneous selection have made substantial contributions. We also found that the variations of environmental conditions along the estuarine locations and among plant species have played a role in shaping the assembly process.

Geographic distance has been found to be crucial in shaping microbial communities in mangrove sediments^4^, ^5^, ^15^. The effect of geographic distance is mainly attributed to dispersal^23^. Dispersal has been found to be important in shaping bacterial^24^, ^25^, archaea^24^, ^26^, and fungal^15^ communities of mangrove sediments. We also found that dispersal limitation played an important role. In different studies, heterogeneous selection^4^, homogeneous selection^5^, homogenizing dispersal^24^, and dispersal limitation^15^, ^26^ have been suggested to dominate mangrove microbial community assembly. We used the most advanced iCAMP method, which resolves the variation of contributions of different phylogenetic bins, revealing that dispersal limitation and homogeneous selection occupy substantial proportions in many bins. The bacterial inhabitants in mangroves may have adapted to endure the highly dynamic environment with disturbances of tidal flow, groundwater discharge, and mud mobility; therefore, they are likely less influenced by environmental factors, which resulted in a very low level of heterogeneous selection in almost all bins^26^.

It has been theoretically^27^, ^28^ and empirically^29^, ^30^ demonstrated that dispersal decreases beta diversity. We found positive correlations between the proportions of dispersal limitation and geographical distances (Figures 4G and S13), suggesting the importance of dispersal limitation in shaping the greater difference among geographic locations. The dispersal and growth of microbes are also influenced by the physicochemical conditions where they inhabit. We found positive correlations between Bray–Curtis distance and environmental distance, and particularly a negative correlation between the alpha diversity of bacterial communities and salinity. Interestingly, although dispersal is usually considered neutral^31^, we found that specific bacterial taxa might have a specific contribution to dispersal limitation. The phylogenetic bins dominated by dispersal limitation were overrepresented by *Patescibacteria*, which are parasitic or symbiotic with hosts due to the lack of several metabolic enzymes^32^.

Homogeneous selection also plays an important role in assembling mangrove bacterial communities. We detected relatively large proportions of homogeneous selection but small proportions of heterogeneous selection, which might be due to mangrove habitats at different places being relatively similar^5^. Nonetheless, mangrove bacterial communities are enriched with specific microbes, which are functionally important for these plants. *Gammaproteobacteria* and *Deltaproteobacteria* are dominant sulfur‐oxidizing bacteria in mangroves^33^, ^34^, which are adaptive to anaerobic and high sulfate conditions^35^. The metabolic versatility of *Chloroflexi* may enable the mangrove plants to live in the fluctuating intertidal environments between land and ocean^36^. Bacteria involved in organic (*Bacteroidales*, *Actinobacteria*) and cellulose (*Prolixibacteraceae*) degradation were enriched in the rhizosphere communities because they can utilize a broad range of root‐derived carbon substrates^37^, ^38^, ^39^. In addition, sulfate‐reducing (*Desulfobulbaceae*)^39^ and sulfide‐oxidizing bacteria (*Ignavibacterium*)^33^, ^35^ are also abundant in rhizosphere communities, indicating a higher sulfur redox and organic‐degrading activity. In contrast, the aerobic nitrifying bacteria (*Nitrospira*), which stick to plant roots and impede root breathing, were reduced in the rhizospheres^40^.

Plants have an influence on rhizosphere bacterial communities. On the one hand, the rhizosphere effect may increase homogeneous selection in rhizosphere bacterial communities^41^. Being the first barrier to root habitat filtration, the rhizosphere promotes an initial substrate‐driven community shift and weakens the influence of environmental stress^41^, ^42^. However, the advanced iCAMP method revealed few differences in homogeneous selection proportions among rhizospheres of different mangroves and the bulk, indicating that the influence of plant presence and plant identity on homogeneous selection is minor. On the other hand, plant roots may exclude bacterial species without appropriate trait combinations for the given abiotic and biotic conditions in the rhizosphere^43^, ^44^. We found a very small proportion of heterogeneous selection in mangrove bacterial communities, indicating a weak effect of rhizosphere habitat filtration.

These ecological processes also influence the bacterial networks, which describe the complex interactions (such as antagonism or cooperation) among species^45^. Plant roots secrete low‐molecular‐weight compounds^34^, ^37^, ^46^ and increase nutrient availability in the rhizosphere microhabitat, allowing the growth of rare taxa and reducing resource competition among bacterias^47^, ^48^. In addition, the communities with a lower environmental gradient may tend to have lower interaction^49^, ^50^, indicated by the observation of a lower environmental gradient (Figure 6A) and a lower level of network complexity in *A. corniculatum* (Figure S2), compared to the other two mangrove species. Complex networks with greater connectivity are generally more robust to environmental perturbations than simple networks with lower connectivity^51^, that is, stronger community stability^52^. In this sense, we speculate that the bacterial communities of *B. sexangula* and *K. obovata* may be more resilient to environmental disturbances than *A. corniculatum*.

In conclusion, we revealed significant differentiation in rhizosphere bacterial community composition, diversity, and interaction networking among the three locations along Dongzhai Harbor, even though the three locations are geographically quite close. In comparison, the rhizosphere bacterial communities differentiated much less among different mangrove species, implying that plant identity has less influence on the microbial community assembly. Such drastic differentiation among geographic locations could be largely attributed to dispersal limitation as well as homogeneous selection. As the iCAMP method indicated, dispersal limitation and homogeneous selection are dominant in some phylogenetic bacterial lineages. Our findings advance the understanding of microbial community assembly in mangrove ecosystems, which could be useful for mangrove conservation and restoration.

## MATERIALS AND METHODS

### Sampling

Dongzhai Harbor is located northeast of Hainan Island. Sanjiang (E110°38′, N19°56′), Yanfeng (E110°35′, N19°58′), and Tashi (E110°33′, N20°06′) are located upstream, midstream, and downstream of Dongzhai Harbor, respectively (Figure 1A). Sanjiang is 5.3 km from Yanfeng; Yanfeng is 6.9 km from Tashi; and Tashi is 12.2 km from Sanjiang. *A. corniculatum*, *B. sexangula*, and *K. obovata* are among the dominant species at all three locations. At each location, we sampled rhizosphere sediments from individual trees of three mangrove species (*A. corniculatum*, *B. sexangula*, and *K. obovata*), which are inundated with an intermediate level of seawater. For each plant species in each location, we sampled five rhizosphere sediment samples from five randomly selected trees at least 5 m apart from each other. At each location, we also collected five bulk sediments that are free of plant roots. Finally, we collected 15 sediment samples for each of the three plants and 15 bulk samples, making up a total of 60 samples.

At each of the three geographic locations, we sampled rhizosphere sediments from five individual trees for each of the three plants, together with five bulk sediments. All the sampled trees were mature individuals. To sample rhizosphere sediments of a tree, we collected plant roots at a depth of 5–15 cm. Rhizosphere sediments tightly adhering to the surface of roots were shaken off and fibrous roots were cut off with an aseptic scissor^53^; these were washed in 20 ml of 0.85% NaCl at 120 rpm for 10 min^54^. To collect one bulk sample, we collected sediments at a depth of 5–15 cm within a quadrat of diameter 2 m. At each geographic location, the five bulk quadrats are at least 5 m apart from each other. Notably, both in sampling rhizosphere sediments of a tree and in sampling a bulk sample, we collected sediments from three rounded areas of diameter 30 cm within the tree or the bulk quadrat. All the sediments sampled from the same tree or a bulk quadrat were pooled as one sample. The sediment samples were packaged in sealed polythene bags and stored on ice before being transferred to a laboratory. In the laboratory, each sample was divided into two subsamples: the first subsample was stored at −80°C before nucleic acid extraction and the second subsample was stored at 4°C before examination of the physicochemical condition.

### Measurement of physicochemical conditions

The salinity of seawater was tested using an optical salinometer (Nanbei). The 1:2.5 sediment/water (dH_2_O) suspensions were shaken for 30 min before measuring the pH using a SevenEasy pH meter (Mettler Toledo). Similarly, the 1:5 sediment/water (dH_2_O) suspensions were shaken for 30 min before measuring the concentration of soluble salt. The salinity of sediment, which was determined by the Seven2Go S3 (Mettler Toledo), was determined to measure the amount of soluble salt. The contents of carbon, nitrogen, hydrogen, and sulfur were measured using a Vario EL cube V3.1.8 in CHNS mode (Elementar Analysensysteme GmbH) after the sediment was air‐dried and filtered using a 200‐mesh screen. We used 20–50 mg sediment of each sample for this measurement. The temperatures of the combustion furnace and the reduction furnace were set to 1150°C and 850°C, respectively. The CO_2_ and SO_2_ column desorption temperatures were set to 240°C and 220°C, respectively. The time duration of oxygenation was 120 s and the total test time for a sample was 10 min. The standard sample used for calibration was sulfanilic acid.

### DNA extraction and 16S rRNA sequencing

For each sediment sample, the total genomic DNA was extracted from 0.5 g of sediment using a HiPure sediment DNA Kit B following the manufacturer's protocol. PCR amplifications were conducted with the primer set 16S v34‐F (CCTACGGGNGGCWGCAG) and 16S v34‐R (GACTACHVGGGTATCTAATCC)^55^, ^56^, which amplified the V3–V4 region of the 16S rRNA gene. The PCR was performed in a 30 µl of reaction mixture according to the following process: initial denaturation at 94°C for 5 min, 25 cycles of denaturation at 94°C for 30 s, annealing at 57°C for 30 s, elongation at 68°C for 30 s, and final elongation at 68°C for 5 min. The amplicons were held at 4°C. All the obtained 16S rRNA amplicons were sequenced on an Illumina HiSeq platform.

### Quality control of sequences and taxonomic classification

The 16S rRNA sequences were analyzed using USEARCH. The paired‐end reads of the 16S V3–V4 region were merged using the FLASH program^57^. Quality control was performed to remove the following reads: (1) reads with quality scores less than 20; (2) reads with lengths less than 400 bp; (3) reads with ambiguous bases; and (4) singletons. The clean sequences were clustered into OTUs at a 97% similarity cutoff using the UPARSE clustering algorithm^58^. Chimeras were removed using the “gold” database (http://drive5.com/uchime/gold.fa). OTU sequences were aligned to the Greengenes database using PyNAST. Nonbacterial 16S sequences were discarded, and unaligned 16S sequences were discarded at a threshold of 75% identity. The taxonomic classification was performed at the 97% similarity level using the Ribosomal Database Project Classifier (version 2.11, http://rdp.cme.msu.edu/) against the SILVA v.132 16S rRNA database at a confidence of 80%^59^. The OTUs of the reads assigned to chloroplasts and archaea were removed. The Shannon and richness indexes were estimated to evaluate alpha diversity by subsampling 7082 reads (the smallest number of sequences with sufficient quality among all 60 samples, Figure S15).

### Analyses of community diversity and structure

Community diversity was quantified using Shannon and richness indexes. Spearman's correlation coefficients were calculated in the psych R library (version 2.2.5). PCoA was performed by the classical multidimensional scaling of beta diversity distance matrices using the *cmdscale* function in R 4.2.0. We also used the *capscale* function in the vegan R package (version 2.6.2) to conduct CAP analysis. We performed the ANOSIM and PERMANOVA based on beta diversity distance matrices to test the dissimilarity of microbial communities among different samples. To compare the dissimilarity of Bray–Curtis distances and environmental distances in different rhizospheres, we performed the analysis of multivariate homogeneity of group dispersions (variances) using the betadisper implemented in the vegan R package (version 2.6.2). The environmental distances were calculated based on scaled Euclidean distances using the vegan R package (version 2.6.2).

The enrichment or depletion of OTUs was analyzed using the edgeR package (version 3.38.4)^60^. We retained the OTUs with an abundance >0.01%. Under the negative binomial generalized linear model, we used the *calcNormFactors* function to obtain normalization factors and the *estimateGLMCommonDisp* and *estimateGLMTagwiseDisp* functions to estimate common and tagwise dispersions. We fitted a negative binomial generalized log‐linear model to the read counts using the *glmFit* function. *p* values were corrected for multiple tests using the approach of Benjamini and Hochberg, with *α* = 0.05^61^. With the bulk samples as controls, we identified the OTUs that are enriched or depleted in the rhizosphere microbial community of each plant species in each location. For each of the three plant species, if an OTU is significantly enriched/depleted at two or three geographic locations, the OTU is considered enriched/depleted in this plant species.

We used a Mantel test, which is based on Spearman's correlation, to test whether the Bray–Curtis distances of bacterial communities are correlated with environmental distances or geographical distances. MRM analysis was performed using the vegan (v. 2.6.2), MuMIn (version 1.46.0), and ecodist (version 2.0.9) R packages. The Hmisc library (version 4.7.0) was selected to evaluate the multicollinearity of physicochemical factors. We conducted RDA using the vegan R package (version 2.6.2) to determine the correlation between bacterial taxa and physicochemical conditions.

### Analyses of assembly mechanisms

We used the NCM to test the potential importance of neutral processes in community assembly, by comparing the detected frequencies of OTUs with their relative abundances in a wider metacommunity^13^. The nonlinear least‐squares method was used in this prediction and the *R* ^2^ value indicates the goodness of fit to the NCM^13^. Under this model, the estimated migration rate evaluates the probability that dispersal from a metacommunity replaces a random loss of an individual in a local community. Larger *m* values indicate that dispersal among microbial communities is less limited^22^, ^62^.

We estimated Levins' niche breadth (B) index according to the formula of Jiao et al.^49^. For a given OTU, a high B‐value indicates wide habitat niche breadth. The *Bcom* was calculated as the average of *B* values from all taxa occurring in one community^63^, ^64^, ^65^. The analysis was conducted using the “niche.width” function in the “spaa” R package (version 0.2.2)^66^.

We also estimated the relative contributions of five different mechanisms, that is, homogeneous selection, heterogeneous selection, dispersal limitation, homogenizing dispersal, and “drift and others,” in assembling microbial communities according to βNTI (beta Nearest Taxon Index) and RCbray (Bray–Curtis‐based Raup Crick metrics). We used the “qpen” method implemented in the iCAMP R package (version 1.5.12) to estimate the βNTI and RCbray values^18^.

We also used the Phylogenetic‐bin‐based null model of the iCAMP R package (version 1.5.12) to infer the proportions of these community assembly mechanisms for individual phylogenetic bacterial taxa^17^. We used the parameters *d*_s_ (phylogenetic signal threshold) = 0.2 and *N*_min_ (minimum number) = 24 in the binning step. In the Mantel test, the phylogenetic signal was considered significant if the Pearson correlation coefficient of the bins was *R* > 0.1 and *p* < 0.05. We conducted this computation using the “icamp.bins” function. The NRI was calculated using the “NRI.p” function. We first conducted iCAMP analysis by pooling samples from the three locations for each plant species (and the bulk), then we applied the analysis by pooling samples of the three plant species for each geographic location, and finally, we computed for each plant species (and the bulk) at each location.

### Analyses of community networks

Network construction was performed following the Molecular Ecological Network Analyses Pipeline (http://ieg4.rccc.ou.edu/mena), which is based on the random matrix theory^67^. We removed the OTUs with abundance <0.01%, retaining a total of 1493 OTUs in this analysis. Only the OTUs occurring in more than 75% of the total samples were used for network computation. The OTU table was tansformed following the centered log‐ratio method, and missing values were filled with the value of 0.01 if paired valid values were available. To perform a comparison, an identical cutoff value of 0.82 was used to construct the networks, and module separation was based on fast greedy modularity optimization. We first constructed networks for rhizosphere bacterial communities for each of the three geographic locations by pooling the rhizosphere sediments of three plants (15 samples for each location). We then constructed networks for each of the three plant species, with 15 samples for each plant (pooling the samples from the three locations). The networks were visualized using Gephi 0.9.1^68^. Nodes in the networks were classified into network hubs, module hubs, connectors, and peripherals following the methods described in Deng et al.^67^.

## AUTHOR CONTRIBUTIONS

Zixiao Guo and Lu Liu conceived the study. Suhua Shi supervised the study. Lu Liu, Nan Wang, and Min Liu collected the samples. Lu Liu, Zixiao Guo, and Nan Wang analyzed the data. Zixiao Guo and Lu Liu wrote the manuscript.

## ETHICS STATEMENT

There was no animal or human experiment involved in this study.

## CONFLICT OF INTERESTS

The authors declare no conflict of interests.

## Supporting information

Supporting information.

## Data Availability

The raw sequence data reported in this article are available in the NCBI Sequence Read Archive under BioProject PRJNA732523. All the codes used in this work are available upon request to the authors.
