# Supplementary material for: Assembly processes underlying bacterial community differentiation among geographically close mangrove forests
Source: mLife. 2023 Mar 23;2(1):73–88. doi: 10.1002/mlf2.12060 (PMC10989747; doi:10.1002/mlf2.12060)
Supplement: Supplementary file 1 — Supporting information. [file MLF2-2-73-s001.docx]

Supplementary information for

**Assembly processes underlying bacterial community differentiation among geographically close mangrove forests**

Authors: Lu Liu, Nan Wang, Min Liu, Zixiao Guo*, Suhua Shi

State Key Laboratory of Biocontrol, Guangdong Key Lab of Plant Resources, Southern Marine Science and Engineering Guangdong Laboratory (Zhuhai), School of Life Sciences, Sun Yat-Sen University, Guangzhou, Guangdong, China.

*Corresponding author:

Zixiao Guo ([*guozx8@mail.sysu.edu.cn*](mailto:guozx8@mail.sysu.edu.cn))

This file contains Tables S1-11 and Figures S1-15.

**Table. S1** Relative abundances of bacterial phyla (class for the *proteobacteria* phylum) in the mangrove rhizosphere and the bulk bacterial communities.

|  | ***Aegiceras corniculatum*** | ***Bruguiera sexangula*** | ***Kandelia obovata*** | **Bulk** |
| --- | --- | --- | --- | --- |
| *Gammaproteobacteria* | 17.50±2.56% | 16.87±1.29% | 18.15±2.48% | 18.68±3.04% |
| *Deltaproteobacteria* | 18.24±2.69% | 16.98±3.19% | 16.14±2.15% | 17.94±4.85% |
| *Bacteroidetes* | 13.47±2.66% | 13.40±2.03% | 13.39±3.60% | 8.63±2.52% |
| *Planctomycetes* | 8.14±1.55% | 7.49±1.89% | 8.73±1.44% | 9.84±3.59% |
| *Chloroflexi* | 4.89±1.24% | 5.69±1.70% | 5.40±1.69% | 7.14±3.01% |
| *Acidobacteria* | 5.94±1.00% | 4.61±0.98% | 5.95±1.16% | 6.02±1.56% |
| *Actinobacteria* | 3.45±0.97% | 5.36±4.32% | 3.54±1.02% | 3.89±3.08% |
| *Patescibacteria* | 4.00±2.19% | 5.14±2.25% | 3.79±1.30% | 2.97±2.23% |
| *Verrucomicrobia* | 3.51±0.80% | 4.32±1.38% | 3.85±0.71% | 3.47±1.82% |
| *Alphaproteobacteria* | 3.63±1.28% | 4.34±1.43% | 3.35±1.14% | 3.37±2.27% |
| Others | 17.24 | 15.79 | 17.69 | 18.04 |

**Table S2.** Permutational multivariate ANOVA (PERMANOVA) to partition sources of rhizosphere bacterial community (n=45) variations among different groupings.

|  | Df | Sums of squares | F. Model | *R*^2^ | *p* |
| --- | --- | --- | --- | --- | --- |
| Computation based on Bray-Curtis distance | | | | | |
| Locations | 2 | 1.644 | 10.078 | 0.256 | *** |
| Plant species | 2 | 0.615 | 3.770 | 0.096 | *** |
| Plant species & Locations | 4 | 1.232 | 3.775 | 0.191 | *** |
| Residuals | 36 | 2.937 |  | 0.457 |  |
| Total | 44 | 6.428 |  | 1 |  |
| Computation based on weighted UniFrac distance | | | | | |
| Locations | 2 | 0.054 | 12.416 | 0.307 | *** |
| Plant species | 2 | 0.018 | 4.091 | 0.101 | *** |
| Plant species & Locations | 4 | 0.026 | 2.975 | 0.147 | *** |
| Residuals | 36 | 0.079 |  | 0.445 |  |
| Total | 44 | 0.177 |  | 1 |  |

*** Represents the corresponding significant value *P*<0.001, ** represents the corresponding significant value *P*<0.01, * represents the corresponding significant value *P*<0.05. Statistical significance (*P*) was calculated based on sequential sums of squares from 9999 permutations.

**Table S3.** Analysis of similarities (ANOSIM) and permutational multivariate ANOVA (PERMANOVA) of the bacterial community composition between the three geographic locations.

|  |  | ANOSIM | | PERMANOVA | | | |
| --- | --- | --- | --- | --- | --- | --- | --- |
|  |  | *R* | *p* | Mean Sqs | F.Model | *R*^2^ | *p* |
| **Computation based on Bray-Curtis distance** | | | | | | | |
| *A. corniculatum* | Upstream vs Midstream | 1 | ** | 0.44 | 5.08 | 0.39 | ** |
|  | Upstream vs Downstream | 1 | ** | 0.39 | 4.98 | 0.38 | ** |
|  | Midstream *vs* Downstream | 0.99 | ** | 0.35 | 4.87 | 0.38 | ** |
| *B. sexangula* | Upstream vs Midstream | 0.58 | ** | 0.35 | 3.74 | 0.32 | ** |
|  | Upstream vs Downstream | 1 | ** | 0.50 | 7.49 | 0.48 | ** |
|  | Midstream *vs* Downstream | 0.91 | * | 0.60 | 5.97 | 0.43 | * |
| *K. obovata* | Upstream vs Midstream | 1 | * | 0.59 | 7.59 | 0.49 | * |
|  | Upstream vs Downstream | 1 | ** | 0.71 | 9.78 | 0.55 | ** |
|  | Midstream *vs* Downstream | 0.98 | ** | 0.38 | 4.45 | 0.36 | * |
| Bulk | Upstream vs Midstream | 0.74 | ** | 0.38 | 3.10 | 0.28 | * |
|  | Upstream vs Downstream | 0.75 | ** | 0.63 | 4.35 | 0.35 | ** |
|  | Midstream *vs* Downstream | 0.44 | * | 0.34 | 2.36 | 0.23 | ** |
| **Computation based on weighted UniFrac distance** | | | | | | | |
| *A. corniculatum* | Upstream vs Midstream | 0.61 | ** | 0.02 | 3.16 | 0.28 | ** |
|  | Upstream vs Downstream | 0.46 | * | 0.01 | 3.75 | 0.32 | * |
|  | Midstream *vs* Downstream | 0.64 | ** | 0.03 | 4.47 | 0.36 | ** |
| *B. sexangula* | Upstream vs Midstream | 0.62 | ** | 0.04 | 6.73 | 0.46 | * |
|  | Upstream vs Downstream | 0.95 | ** | 0.06 | 22.03 | 0.73 | ** |
|  | Midstream *vs* Downstream | 0.41 | ** | 0.02 | 3.85 | 0.33 | * |
| *K. obovata* | Upstream vs Midstream | 0.76 | * | 0.03 | 8.19 | 0.51 | ** |
|  | Upstream vs Downstream | 0.86 | ** | 0.04 | 17.16 | 0.68 | ** |
|  | Midstream *vs* Downstream | 0.56 | ** | 0.02 | 4.01 | 0.33 | ** |
| Bulk | Upstream vs Midstream | 0.34 | * | 0.01 | 2.62 | 0.25 | * |
|  | Upstream vs Downstream | 0.69 | * | 0.02 | 5.66 | 0.41 | * |
|  | Midstream *vs* Downstream | 0.30 | * | 0.01 | 2.82 | 0.26 | * |

**Table S4.** Analysis of similarities (ANOSIM) and permutational multivariate ANOVA (PERMANOVA) of the bacterial community composition between bulk and rhizosphere sediments

|  |  | ANOSIM | | PERMANOVA | | | |
| --- | --- | --- | --- | --- | --- | --- | --- |
|  |  | *R* | *p* | Mean Sqs | F.Model | *R*^2^ | *p* |
| Computation based on Bray-Curtis distance | *A. corniculatum vs* Bulk | 0.43 | *** | 0.67 | 4.38 | 0.14 | *** |
|  | *B. sexangula vs* Bulk | 0.54 | *** | 0.96 | 5.88 | 0.17 | *** |
|  | *K. obovata vs* Bulk | 0.29 | ** | 0.43 | 2.62 | 0.09 | ** |
|  | *A. corniculatum vs B. sexangula* | 0.11 | * | 0.28 | 2.08 | 0.07 | * |
|  | *A. corniculatum vs K. obovata* | 0.06 | - | 0.20 | 1.47 | 0.05 | - |
|  | *B. sexangula vs K. obovata* | 0.17 | ** | 0.44 | 3.05 | 0.10 | ** |
|  |  |  |  |  |  |  |  |
| Computation based on Weighted UniFrac distance | *A. corniculatum vs* Bulk | 0.69 | *** | 0.09 | 14.16 | 0.34 | *** |
|  | *B. sexangula vs* Bulk | 0.43 | *** | 0.05 | 6.38 | 0.19 | ** |
|  | *K. obovata vs* Bulk | 0.48 | *** | 0.06 | 10.51 | 0.27 | *** |
|  | *A. corniculatum vs B. sexangula* | 0.14 | * | 0.03 | 4.04 | 0.13 | * |
|  | *A. corniculatum vs K. obovata* | 0.02 | - | 0.01 | 0.90 | 0.03 | - |
|  | *B. sexangula vs K. obovata* | 0.13 | * | 0.03 | 3.25 | 0.10 | * |

**Table S5.** Key topological features of bacterial community networks in different geographical locations.

|  | Network indexes | Upstream | Midstream | Downstream |
| --- | --- | --- | --- | --- |
| Empirical networks | Similarity threshold | 0.820 | 0.820 | 0.820 |
|  | *R*^2^ of power law | 0.818 | 0.963 | 0.844 |
|  | Average connectivity (avgK) | 11.564 | 2.936 | 4.035 |
|  | Average path distance (GD) | 3.722 | 5.235 | 4.798 |
|  | Harmonic geodesic distance (HD) | 3.102 | 4.257 | 3.888 |
|  | Average clustering coefficient (avgCC) | 0.163 | 0.115 | 0.092 |
|  | Connectedness (Con) | 0.782 | 0.386 | 0.554 |
|  | Density | 0.023 | 0.008 | 0.012 |
|  | Modularity | 0.315 | 0.736 | 0.574 |
| Random networks | HD | 2.677±0.015 | 3.950±0.053 | 3.348 ±0.034 |
|  | avgCC | 0.135±0.006 | 0.016±0.005 | 0.036±0.005 |
|  | Modularity | 0.208±0.003 | 0.617±0.007 | 0.473±0.006 |

**Table S6.** Taxonomic information of the nodes in the bacterial community networks of the three geographic locations.

|  | Upstream | Midstream | Downstream |
| --- | --- | --- | --- |
| *Acidobacteria* | 7.00% | 7.75% | 8.36% |
| *Actinobacteria* | 3.20% | 2.41% | 3.17% |
| *Alphaproteobacteria ^*^* | 5.20% | 3.21% | 3.75% |
| *Bacteroidetes* | 11.80% | 12.57% | 12.68% |
| *Chloroflexi* | 3.40% | 3.21% | 3.75% |
| *Deltaproteobacteria ^*^* | 17.00% | 12.83% | 15.27% |
| *Gammaproteobacteria ^*^* | 15.80% | 15.51% | 17.29% |
| *Gemmatimonadetes* | 5.00% | 4.55% | 3.17% |
| *Kiritimatiellaeota* | 2.00% | 2.41% | 3.75% |
| *Nitrospirae* | 2.20% | 2.67% | 0.58% |
| *Patescibacteria* | 2.60% | 4.55% | 2.88% |
| *Planctomycetes* | 10.40% | 10.70% | 8.07% |
| *Verrucomicrobia* | 4.20% | 5.88% | 5.19% |
| Others | 10.20% | 11.76% | 12.10% |

^*^These three are classes of the phylum *Proteobacteria*.

**Table S7.** Relative importance of each ecological process in different mangrove sediments

|  | Heterogeneous selection | Homogeneous selection | Dispersal limitation | Homogenizing dispersal | Drift and others |
| --- | --- | --- | --- | --- | --- |
| *A. corniculatum* | 0.64% | 12.36% | 11.56% | 6.81% | 68.64% |
| *B. sexangula* | 0.61% | 14.44% | 16.66% | 8.16% | 60.13% |
| *K. obovata* | 0.49% | 16.06% | 16.26% | 6.00% | 61.18% |
| Bulk | 1.01% | 15.00% | 28.82% | 4.94% | 50.23% |

**Table S8**. Bins dominated by dispersal limitation or homogeneous selection

| Group | Bins | | | | | | | |
| --- | --- | --- | --- | --- | --- | --- | --- | --- |
| **Bins dominated by dispersal limitation** | | | | | | | | |
| *A. corniculatum* | Bin10 | Bin115 | Bin116 | Bin31 | Bin5 | Bin54 | Bin71 | Bin83 |
| *B. sexangula* | Bin106 | Bin115 | Bin116 | Bin117 | Bin123 | Bin23 | Bin24 | Bin27 |
|  | Bin29 | Bin30 | Bin33 | Bin36 | Bin71 | Bin83 |  |  |
| *K. obovata* | Bin10 | Bin115 | Bin36 | Bin60 | Bin71 | Bin83 | Bin91 | Bin98 |
| Bulk | Bin1 | Bin103 | Bin104 | Bin111 | Bin115 | Bin123 | Bin124 | Bin125 |
|  | Bin13 | Bin18 | Bin24 | Bin25 | Bin27 | Bin3 | Bin32 | Bin35 |
|  | Bin37 | Bin40 | Bin41 | Bin44 | Bin47 | Bin5 | Bin6 | Bin63 |
|  | Bin71 | Bin72 | Bin73 | Bin77 | Bin79 | Bin9 | Bin90 | Bin91 |
|  | Bin92 | Bin95 |  |  |  |  |  |  |
| **Bins dominated by Homogeneous selection** | | | | | | | | |
| *A. corniculatum* | Bin114 | Bin17 | Bin34 | Bin51 | Bin59 | Bin75 | Bin80 | Bin86 |
|  | Bin92 |  |  |  |  |  |  |  |
| *B. sexangula* | Bin114 | Bin125 | Bin17 | Bin22 | Bin34 | Bin4 | Bin59 | Bin75 |
|  | Bin80 | Bin92 |  |  |  |  |  |  |
| *K. obovata* | Bin108 | Bin114 | Bin17 | Bin34 | Bin4 | Bin42 | Bin49 | Bin51 |
|  | Bin59 | Bin75 | Bin8 | Bin80 | Bin86 | Bin92 |  |  |
| Bulk | Bin114 | Bin119 | Bin17 | Bin42 | Bin49 | Bin51 | Bin59 | Bin8 |
|  | Bin85 |  |  |  |  |  |  |  |

**Table S9.** Physicochemical properties of all samples.

| ID | Sediment | Location | pH | Salinity (PSU) | Moisture(%) | Total carbon (%) | Total hydrogen (%) | Total nitrogen (%) | Total sulfur (%) | Carbon/nitrogen |
| --- | --- | --- | --- | --- | --- | --- | --- | --- | --- | --- |
| 1 | *A. corniculatum* | Upstream | 6.53 | 2.53 | 97.30 | 3.77 | 1.05 | 0.21 | 0.31 | 17.95 |
| 2 | *A. corniculatum* | Upstream | 6.22 | 2.63 | 81.14 | 4.35 | 1.13 | 0.24 | 0.26 | 18.13 |
| 3 | *A. corniculatum* | Upstream | 6.55 | 2.4 | 114.44 | 3.6 | 1.09 | 0.2 | 0.15 | 18 |
| 4 | *A. corniculatum* | Upstream | 6.66 | 2.87 | 122.18 | 5.3 | 1.3 | 0.27 | 0.42 | 19.63 |
| 5 | *A. corniculatum* | Upstream | 6.22 | 2.2 | 138.22 | 4.99 | 1.24 | 0.26 | 0.24 | 19.19 |
| 6 | *B. sexangula* | Upstream | 6.16 | 1.91 | 79.78 | 3.71 | 1.06 | 0.22 | 0.29 | 16.86 |
| 7 | *B. sexangula* | Upstream | 6.31 | 1.88 | 78.18 | 3.13 | 1 | 0.17 | 0.16 | 18.41 |
| 8 | *B. sexangula* | Upstream | 6.21 | 2.74 | 82.51 | 3.67 | 1.08 | 0.21 | 0.3 | 17.48 |
| 9 | *B. sexangula* | Upstream | 6.2 | 2.25 | 73.31 | 2.97 | 0.97 | 0.17 | 0.2 | 17.47 |
| 10 | *B. sexangula* | Upstream | 6.51 | 2.05 | 87.78 | 4.39 | 1.21 | 0.24 | 0.4 | 18.29 |
| 11 | *K. obovata* | Upstream | 6.87 | 2.18 | 62.85 | 1.34 | 0.72 | 0.1 | 0.11 | 13.40 |
| 12 | *K. obovata* | Upstream | 6.84 | 1.97 | 59.54 | 1.2 | 0.59 | 0.08 | 0.14 | 15 |
| 13 | *K. obovata* | Upstream | 6.97 | 1.99 | 68.00 | 1.27 | 0.69 | 0.1 | 0.1 | 12.70 |
| 14 | *K. obovata* | Upstream | 7.01 | 2.25 | 63.20 | 1.16 | 0.67 | 0.09 | 0.09 | 12.89 |
| 15 | *K. obovata* | Upstream | 7.05 | 1.59 | 54.78 | 1.02 | 0.53 | 0.08 | 0.09 | 12.75 |
| 16 | Bulk | Upstream | 6.89 | 1.14 | 60.60 | 1.14 | 0.65 | 0.09 | 0.09 | 12.67 |
| 17 | Bulk | Upstream | 6.85 | 1.77 | 46.79 | 0.86 | 0.58 | 0.08 | 0.08 | 10.75 |
| 18 | Bulk | Upstream | 7.02 | 1.95 | 50.00 | 0.85 | 0.52 | 0.07 | 0.07 | 12.14 |
| 19 | Bulk | Upstream | 7.17 | 1.12 | 42.86 | 1.17 | 1.03 | 0.1 | 0.07 | 11.7 |
| 20 | Bulk | Upstream | 6.81 | 1.35 | 44.72 | 1.39 | 0.96 | 0.11 | 0.1 | 12.64 |
| 21 | *A. corniculatum* | Midstream | 6.75 | 2.55 | 75.18 | 4.05 | 1.31 | 0.23 | 0.42 | 17.61 |
| 22 | *A. corniculatum* | Midstream | 6.7 | 2.72 | 88.09 | 3.89 | 1.31 | 0.25 | 0.47 | 15.56 |
| 23 | *A. corniculatum* | Midstream | 6.8 | 2.36 | 100.38 | 4.48 | 1.26 | 0.24 | 0.55 | 18.67 |
| 24 | *A. corniculatum* | Midstream | 6.66 | 2.85 | 90.10 | 3.82 | 1.32 | 0.25 | 0.39 | 15.28 |
| 25 | *A. corniculatum* | Midstream | 6.49 | 2.73 | 80.84 | 3.83 | 1.29 | 0.23 | 0.26 | 16.65 |
| 26 | *B. sexangula* | Midstream | 6.2 | 2.6 | 91.94 | 4.68 | 1.35 | 0.24 | 0.42 | 19.50 |
| 27 | *B. sexangula* | Midstream | 6.41 | 2.86 | 103.97 | 4.14 | 1.18 | 0.22 | 0.83 | 18.82 |
| 28 | *B. sexangula* | Midstream | 6.31 | 2.07 | 112.32 | 4.34 | 1.31 | 0.21 | 0.38 | 20.67 |
| 29 | *B. sexangula* | Midstream | 6.61 | 3.36 | 78.71 | 4.48 | 1.31 | 0.24 | 0.31 | 18.67 |
| 30 | *B. sexangula* | Midstream | 6.63 | 2.62 | 99.49 | 4.71 | 1.33 | 0.27 | 0.34 | 17.44 |
| 31 | *K. obovata* | Midstream | 6.42 | 2.55 | 93.33 | 4.51 | 1.32 | 0.25 | 0.57 | 18.04 |
| 32 | *K. obovata* | Midstream | 6.57 | 2.31 | 97.75 | 5.53 | 1.42 | 0.27 | 0.59 | 20.48 |
| 33 | *K. obovata* | Midstream | 6.4 | 3.1 | 86.07 | 5.07 | 1.43 | 0.28 | 0.58 | 18.11 |
| 34 | *K. obovata* | Midstream | 6.3 | 2.51 | 98.47 | 4.2 | 1.18 | 0.23 | 0.53 | 18.26 |
| 35 | *K. obovata* | Midstream | 6.42 | 2.64 | 86.71 | 4.74 | 1.22 | 0.22 | 1.12 | 21.55 |
| 36 | Bulk | Midstream | 6.67 | 2.61 | 100.56 | 3.09 | 1.06 | 0.18 | 0.98 | 17.17 |
| 37 | Bulk | Midstream | 6.79 | 2.69 | 90.78 | 2.78 | 0.99 | 0.17 | 0.97 | 16.35 |
| 38 | Bulk | Midstream | 7 | 2.64 | 102.13 | 3.38 | 1.16 | 0.21 | 0.8 | 16.10 |
| 39 | Bulk | Midstream | 6.48 | 1.99 | 75.44 | 3.02 | 1.25 | 0.18 | 0.36 | 16.78 |
| 40 | Bulk | Midstream | 6.38 | 2.18 | 72.41 | 2.61 | 1.25 | 0.17 | 0.54 | 15.35 |
| 41 | *A. corniculatum* | Downstream | 6.48 | 2.64 | 69.43 | 3.22 | 0.69 | 0.19 | 0.53 | 16.95 |
| 42 | *A. corniculatum* | Downstream | 6.43 | 1.95 | 69.19 | 2.68 | 0.6 | 0.16 | 0.36 | 16.75 |
| 43 | *A. corniculatum* | Downstream | 6.6 | 2.29 | 68.47 | 3.59 | 0.79 | 0.2 | 0.51 | 17.95 |
| 44 | *A. corniculatum* | Downstream | 6.13 | 2.65 | 71.73 | 2.82 | 0.63 | 0.16 | 0.46 | 17.63 |
| 45 | *A. corniculatum* | Downstream | 6 | 2.09 | 54.92 | 2.98 | 0.65 | 0.18 | 0.43 | 16.56 |
| 46 | *B. sexangula* | Downstream | 6.34 | 3.42 | 140.06 | 8.1 | 1.33 | 0.36 | 0.94 | 22.50 |
| 47 | *B. sexangula* | Downstream | 6.17 | 2.93 | 110.21 | 8.48 | 1.37 | 0.38 | 0.96 | 22.32 |
| 48 | *B. sexangula* | Downstream | 6.26 | 2.77 | 141.92 | 6.86 | 1.17 | 0.31 | 0.71 | 22.13 |
| 49 | *B. sexangula* | Downstream | 6.27 | 2.79 | 134.68 | 6.48 | 1.14 | 0.31 | 0.94 | 20.90 |
| 50 | *B. sexangula* | Downstream | 6.37 | 3.39 | 154.08 | 7.89 | 1.29 | 0.36 | 1.12 | 21.92 |
| 51 | *K. obovata* | Downstream | 6.61 | 2.02 | 79.35 | 2.42 | 0.48 | 0.12 | 0.37 | 20.17 |
| 52 | *K. obovata* | Downstream | 6.38 | 2.71 | 83.11 | 3.5 | 0.63 | 0.19 | 0.41 | 18.42 |
| 53 | *K. obovata* | Downstream | 5.91 | 2.1 | 81.56 | 4.91 | 0.89 | 0.25 | 0.62 | 19.64 |
| 54 | *K. obovata* | Downstream | 6.05 | 2.66 | 88.60 | 6.37 | 1.05 | 0.25 | 0.68 | 25.48 |
| 55 | *K. obovata* | Downstream | 6.42 | 2.24 | 62.65 | 3.36 | 0.68 | 0.18 | 0.45 | 18.67 |
| 56 | Bulk | Downstream | 6.94 | 2.01 | 58.69 | 2.27 | 0.66 | 0.12 | 0.92 | 18.92 |
| 57 | Bulk | Downstream | 6.85 | 2.07 | 65.13 | 2.05 | 0.64 | 0.12 | 0.74 | 17.08 |
| 58 | Bulk | Downstream | 6.79 | 1.68 | 49.61 | 1.64 | 0.6 | 0.11 | 0.67 | 14.91 |
| 59 | Bulk | Downstream | 6.7 | 1.91 | 70.94 | 2.42 | 0.78 | 0.15 | 0.49 | 16.13 |
| 60 | Bulk | Downstream | 6.14 | 2.58 | 36.99 | 1.87 | 0.67 | 0.12 | 0.70 | 15.58 |

**Table S10.** Correlations between Bray-Curtis distances and environmental distances (or geographical distances) determined using Mantel and partial Mantel test.

|  |  | *A. corniculatum* | | *B. sexangula* | | *K. obovata* | | Bulk | |
| --- | --- | --- | --- | --- | --- | --- | --- | --- | --- |
|  | Controlling for: | *R* | *p* | *R* | *p* | *R* | *p* | *R* | *p* |
| Environmental distance |  | 0.425 | 7e-04 | 0.482 | 3e-04 | 0.726 | 1e-04 | 0.349 | 0.003 |
| Geographical distance |  | 0.662 | 2e-04 | 0.563 | 3e-04 | 0.809 | 1e-04 | 0.637 | 1e-04 |
| Environmental distance | Geographical distance | 0.033 | 0.368 | 0.033 | 0.385 | 0.668 | 1e-04 | 0.136 | 0.122 |
| Geographical distance | Environmental distance | 0.562 | 2e-04 | 0.334 | 0.003 | 0.771 | 1e-04 | 0.580 | 1e-04 |

**Table S11.** Multiple regression analysis on matrices (MRM) for each of the four sediment types and all sediments.

|  | All  *R*^2^=0.32, *p<0.001* | | 1. *corniculatum*   *R*^2^=0.39, *p<0.001* | | 1. *sexangula*   *R*^2^=0.36, *p<0.001* | | 1. *obovata*   *R*^2^=0.53, *p<0.001* | | Bulk  *R*^2^=0.13, *p=0.086* | |
| --- | --- | --- | --- | --- | --- | --- | --- | --- | --- | --- |
|  | r | *p* | r | *p* | r | *p* | r | *p* | r | *p* |
| pH | 0.055 | 1.0e-4 | 0.004 | 0.781 | 0.017 | 0.478 | - | - | 0.026 | 0.463 |
| Salinity | 0.006 | 0.628 | 0.002 | 0.846 | - | - | 0.006 | 0.651 | 0.023 | 0.557 |
| Moisture | - | - | 0.059 | 0.002 | 0.081 | 0.011 | 0.011 | 0.544 | - | - |
| Total carbon (%) | 0.048 | 0.005 | 0.002 | 0.902 | 0.051 | 0.090 | 0.106 | 0.006 | 0.013 | 0.726 |
| Total hydrogen (%) | - | - | - | - | 0.021 | 0.309 | - | - | - | - |
| Total nitrogen (%) | - | - | - | - | - | - | - | - | - | - |
| Total sulfur (%) | 0.053 | 1.0e-4 | 0.057 | 0.002 | - | - | - | - | 0.093 | 0.007 |
| Carbon/nitrogen | 0.015 | 0.383 | 0.013 | 0.375 | - | - | 0.055 | 0.023 | - | - |


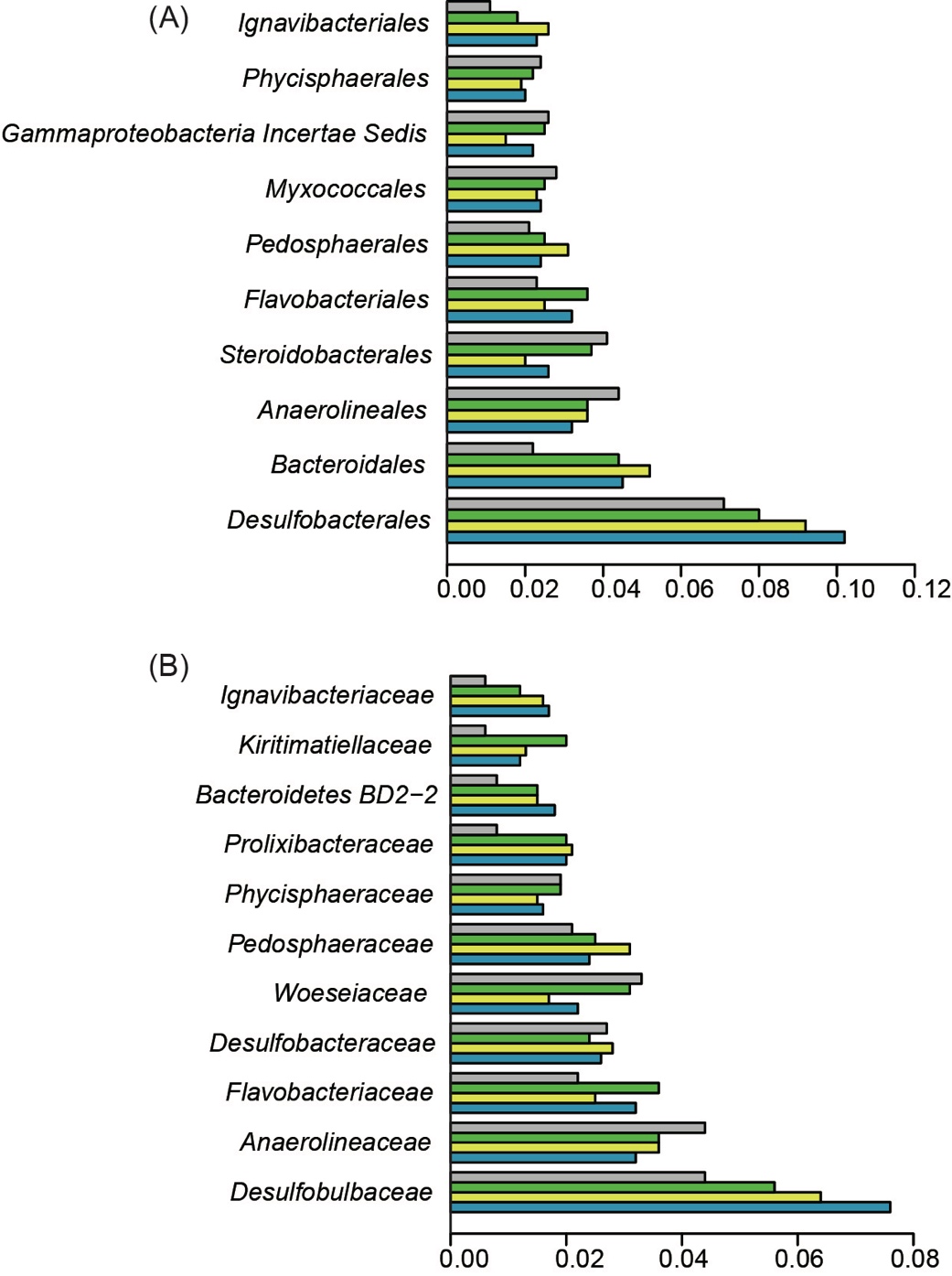


**Fig. S1** Relative abundances of bacterial orders (A) and families (B) in the mangrove rhizosphere and the bulk bacterial communities. Blue, yellow and green indicate the rhizospheres of *Aegiceras corniculatum,* *Bruguiera sexangula,* and *Kandelia obovata*, respectively. Grey indicates the bulk sediment.


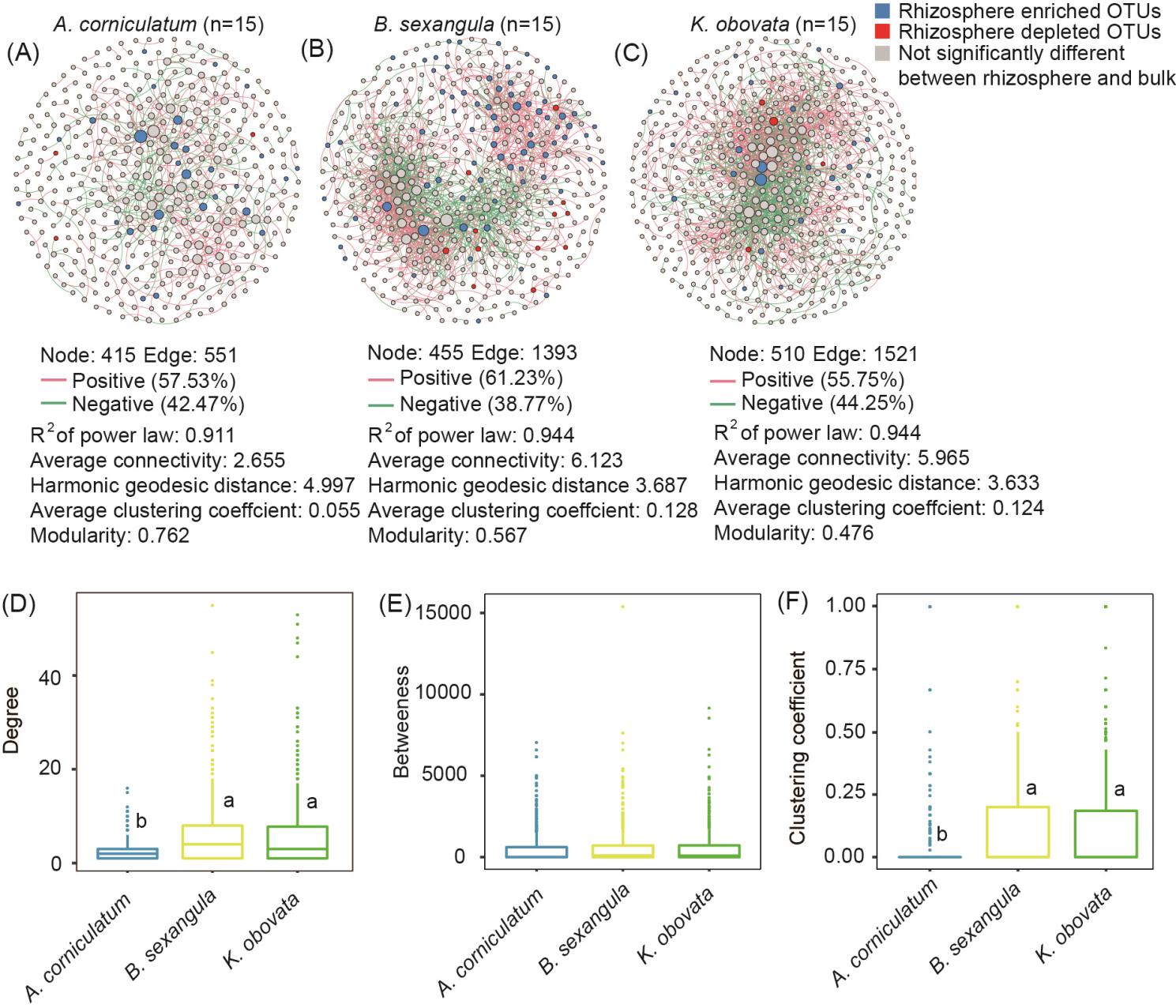


**Fig. S2** Interaction networks of the mangrove rhizosphere bacterial communities. (A-C) Graphic overview of the bacterial networks of the three mangrove rhizosphere sediments. Node size is proportional to node connectivity. Node colors indicate whether the corresponding OTU is enriched or depleted in comparison to bulk samples. Line colors indicate positive (red) or negative (green) correlations. (D-F) Degree, betweenness, and clustering coefficient of each node in the networks of the three mangrove species (Tukey-HSD test).


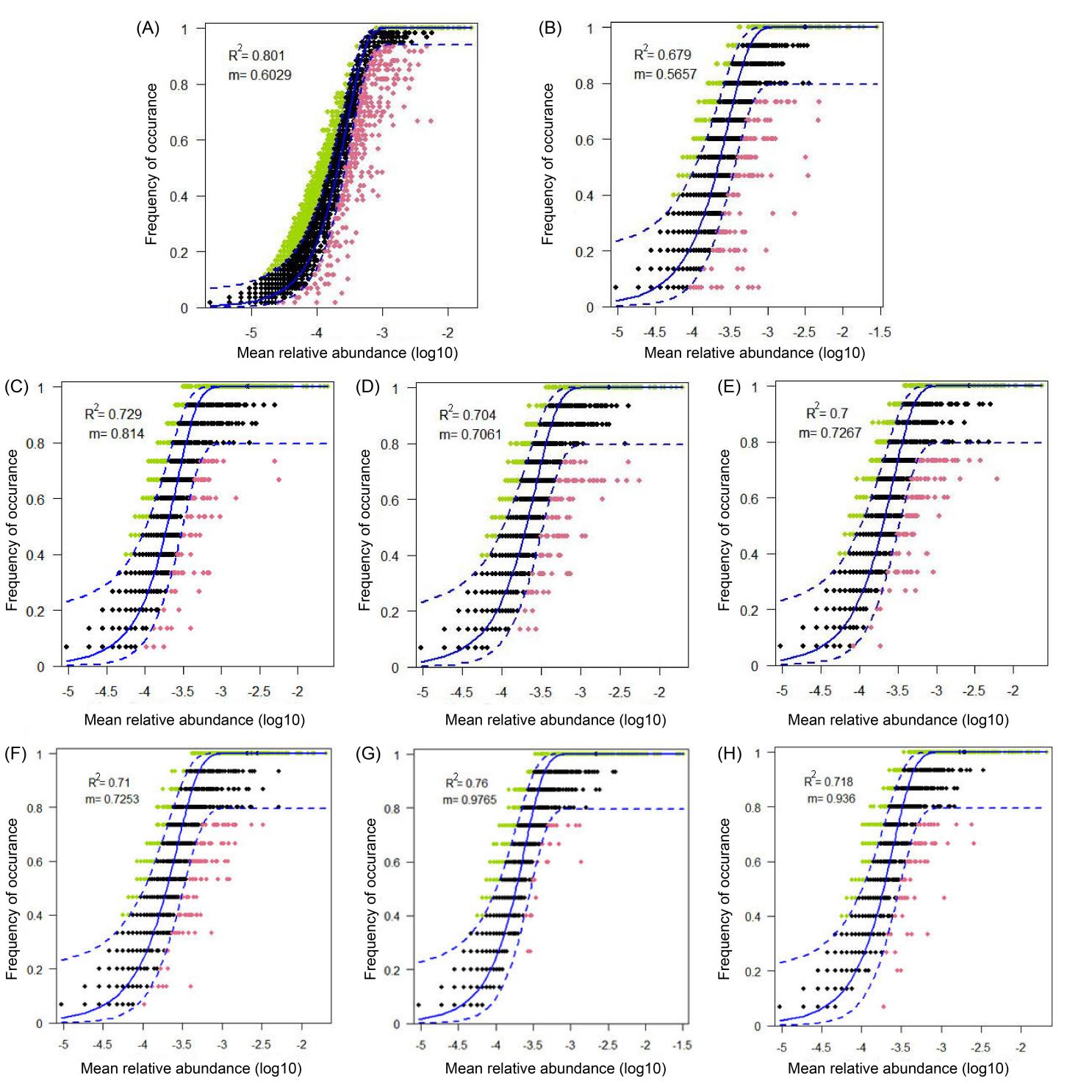


**Fig. S3** Test of the neutral community model (NCM). Occurrence frequencies of OTUs in the bacterial communities of all (n=60) (A), bulk (n=15) (B), *A*. corniculatum (n=15) (C), *B*. sexangula (n=15) (D), *K. obovata* (n=15) (E), upstream (n=15, only rhizosphere samples were included) (F), midstream (n=15, only rhizosphere samples were included) (G) and downstream (n=15, only rhizosphere samples were included) (H) sediments, respectively. The solid blue lines indicate the values predicted based on NCM. The dashed blue lines indicate 95% confidence intervals. The OTUs with occurrence frequency within the 95% intervals are in black, while those with higher or lower frequency are colored in green or red. *R*^2^ evaluates the fit to the NCM. m indicates migration.

**
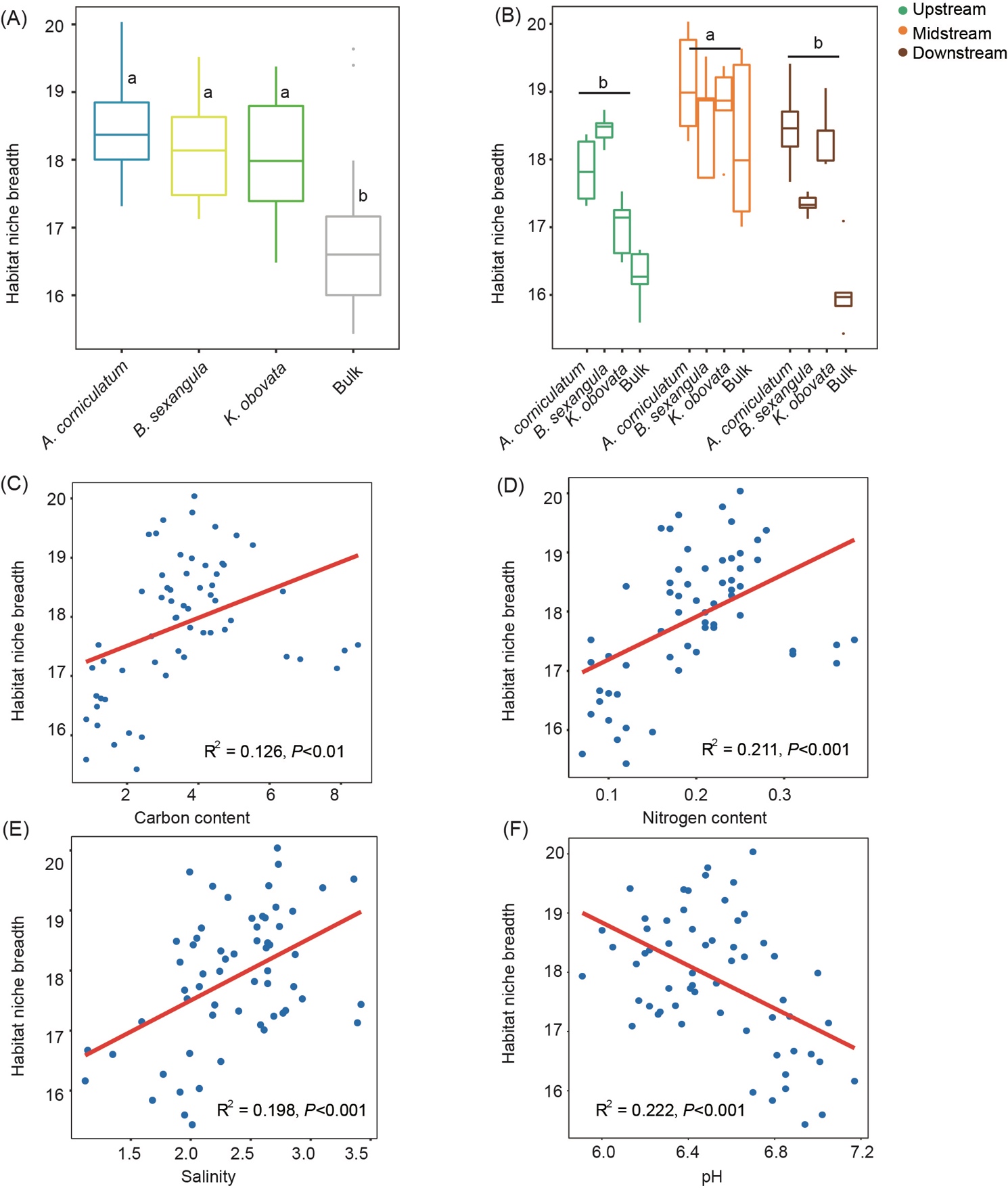
**

**Fig. S4** Habitat niche breadths (*Bcom*) of different samples. (A) Distribution of *Bcom* values of different species. (B) Distribution of *Bcom* values of different geographic Locations. The lowercase letters indicate significant levels (Tukey-HSD test). (C-F) Correlations between *Bcom* values and environmental conditions: sediment total carbon (C), nitrogen content (D), salinity (E), and pH (F).


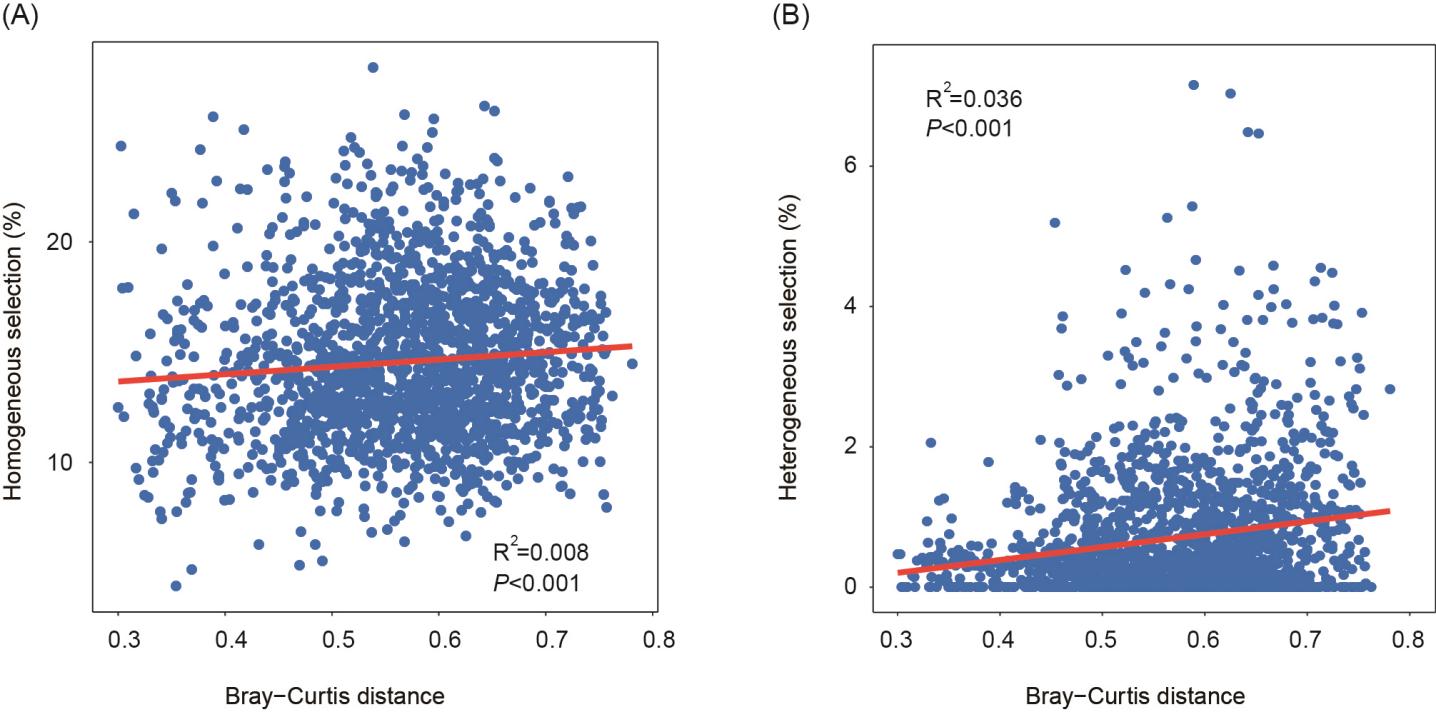


**Fig. S5** Correlation between Bray-Curtis distance with the relative importance of (A) homogeneous selection and (B) heterogeneous selection


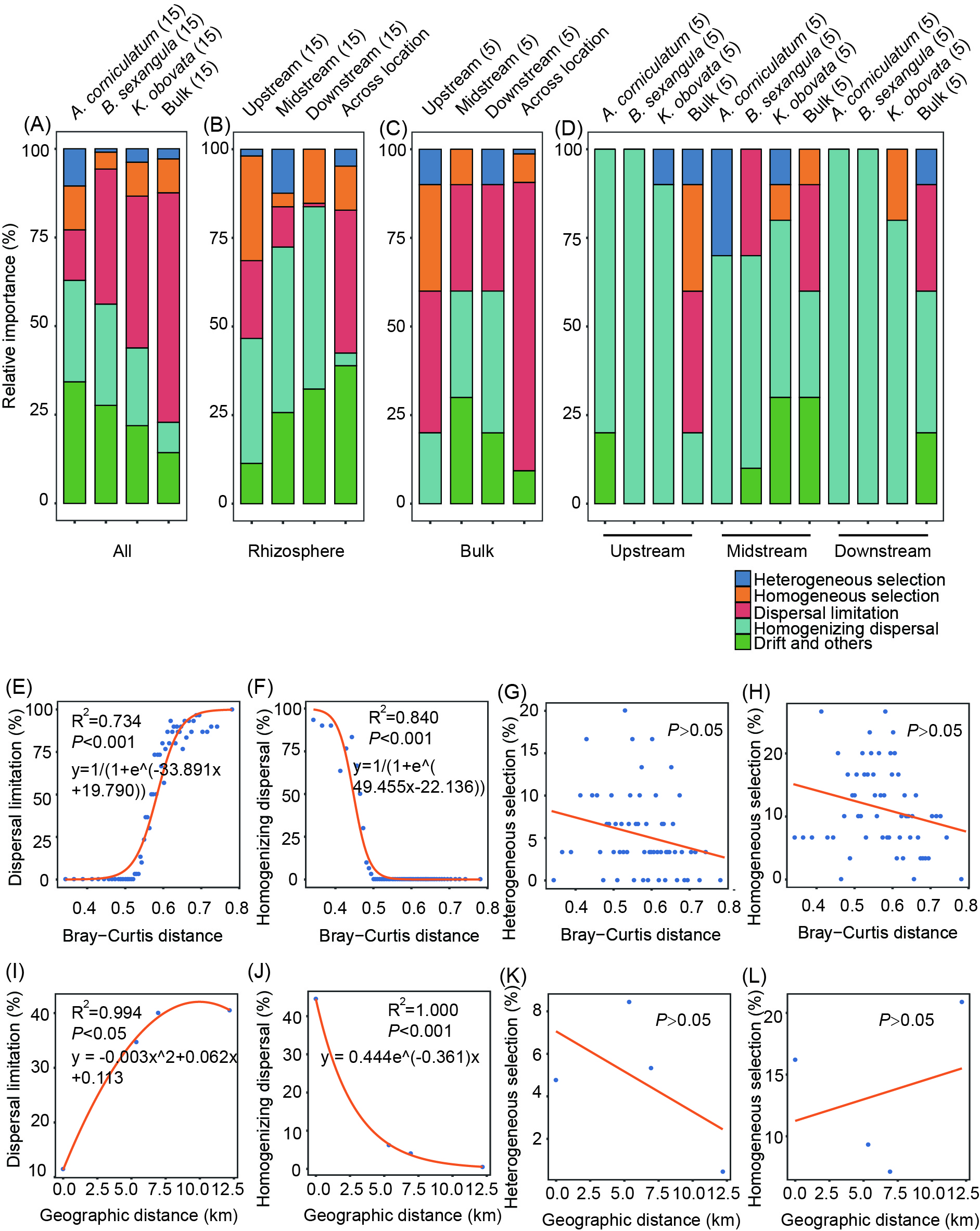


**Fig. S6** Mechanisms of bacterial community assembly in mangroves based on βNTI (beta Nearest Taxon Index) and RCbray (Bray Curtis based Raup Crick metrics) values. (A-D) Proportions of different assembly mechanisms computed for the four sediment types (A), rhizosphere sediments of the three geographic locations (B), bulk sediments of the three geographic locations (C), and each sediment type at each location (D). Notably, “across location” indicates the computation was conducted by combing all three locations. (E-H) Correlations of the relative importance of dispersal limitation, homogenizing dispersal, heterogeneous selection, and homogeneous selection with Bray-Curtis distances. In the calculations of E-H, we first ranked Bray-Curtis distances from small to large, then we counted the proportions of different mechanisms by every 30 distance values. (I-L) Correlations of the relative importance of dispersal limitation, homogenizing dispersal, heterogeneous selection, and homogeneous selection with geographic distances.


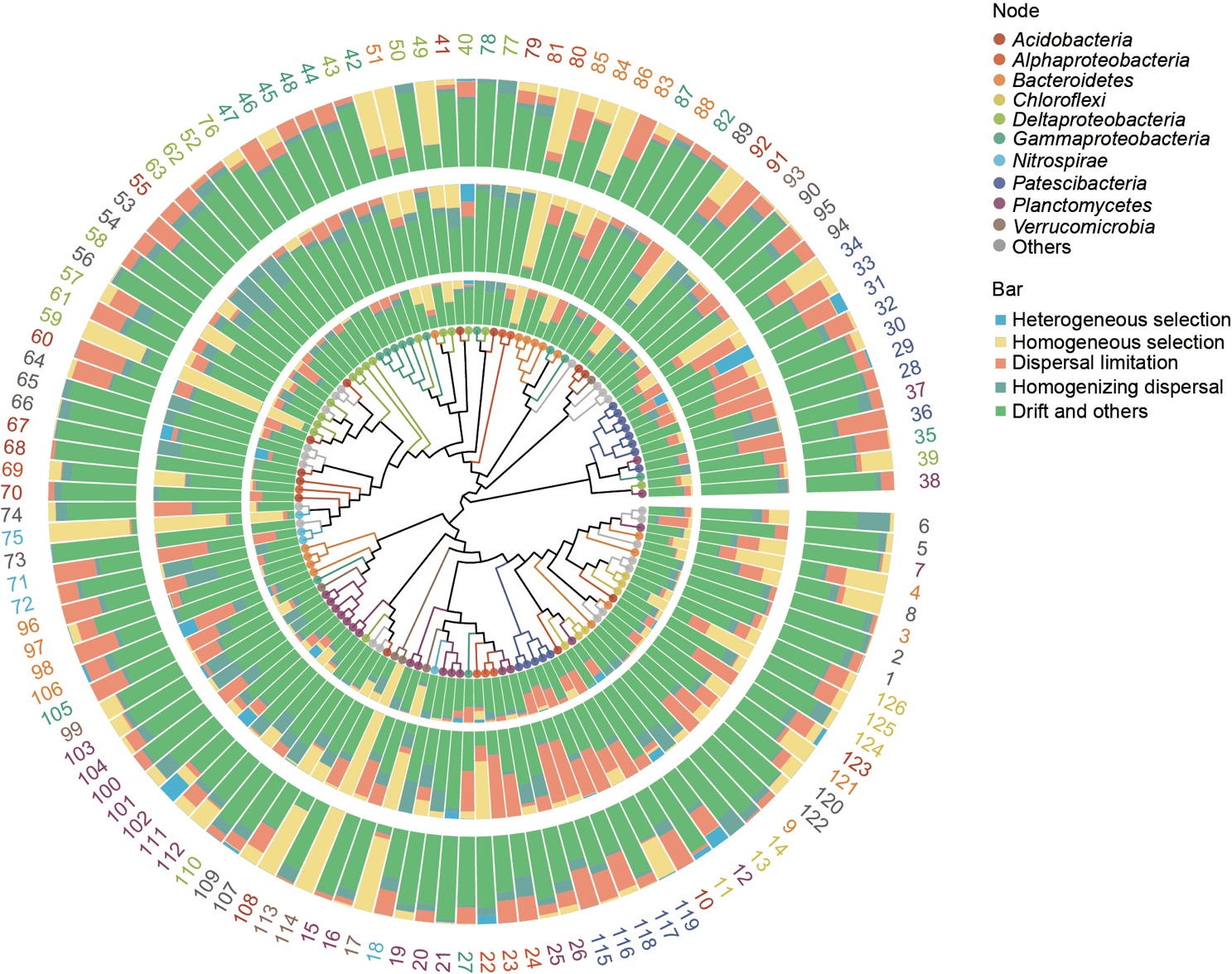


**Fig. S7** Variation of assembly mechanisms across different phylogenetic bins in different mangrove species. At the center is a phylogenetic tree. From inner to outer, the three tracks present the relative importance of different assembly mechanisms in each bin in rhizosphere samples of *A. corniculatum*, *B. sexangula*, and *K. obovata*


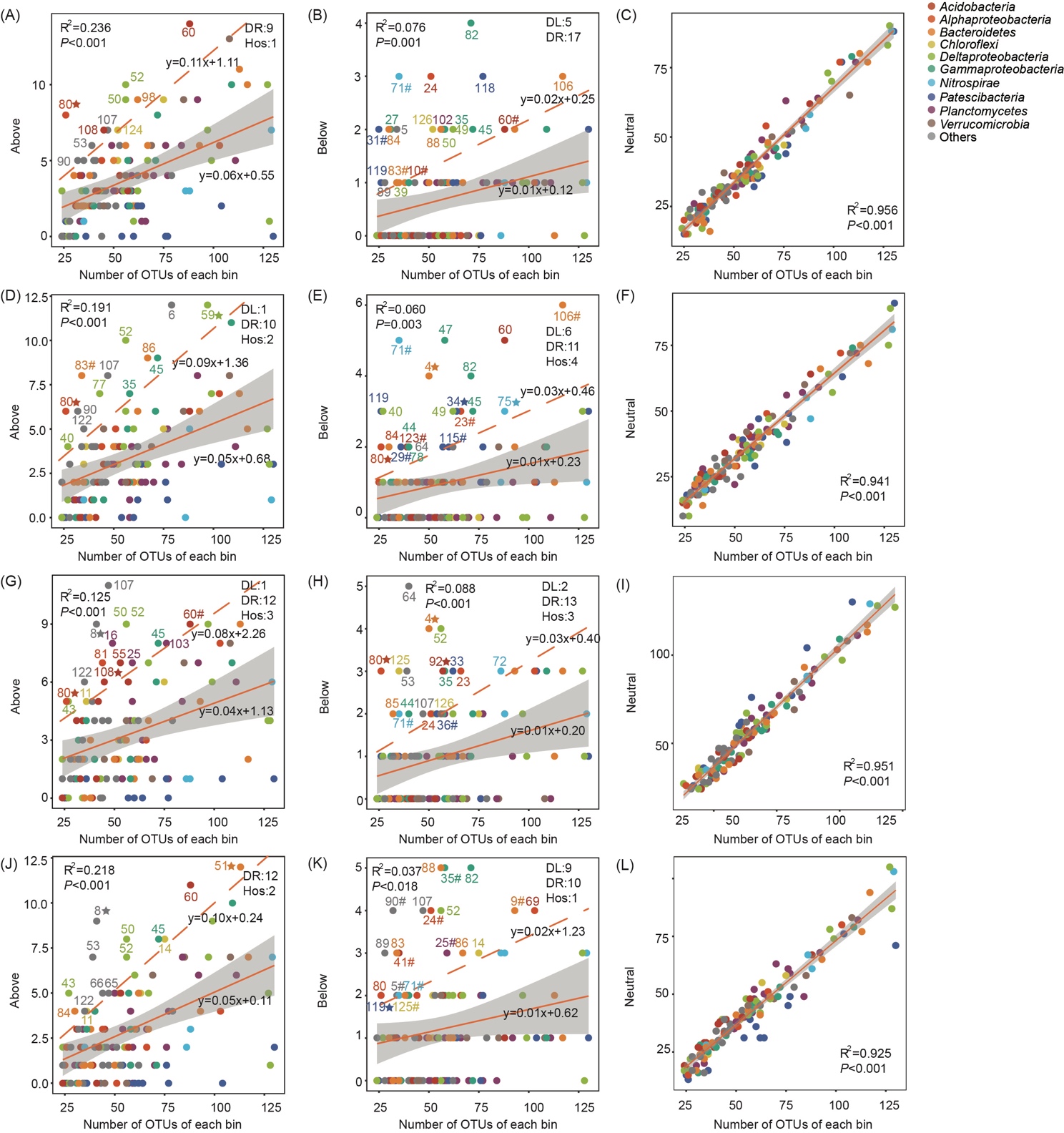


**Fig. S8** Correlations between the numbers of OTUs above, below, or within the NCM expectation and the iCAMP bin sizes (numbers of OTUs in iCAMP bins). We highlighted the bins with significantly more OTUs than the predicted value from bin size. (A-C) *Aegiceras corniculatum*; (D-F) *Bruguiera sexangula*; (G-I) *Kandelia obovata*; (J-L) bulk samples. “#” represents dispersal limitation dominated bins, “★” represents homogeneous selection-dominated bins. “DL” represents dispersal limitation; “DR” represent “drift and others”; “Hos” represents homogeneous selection.


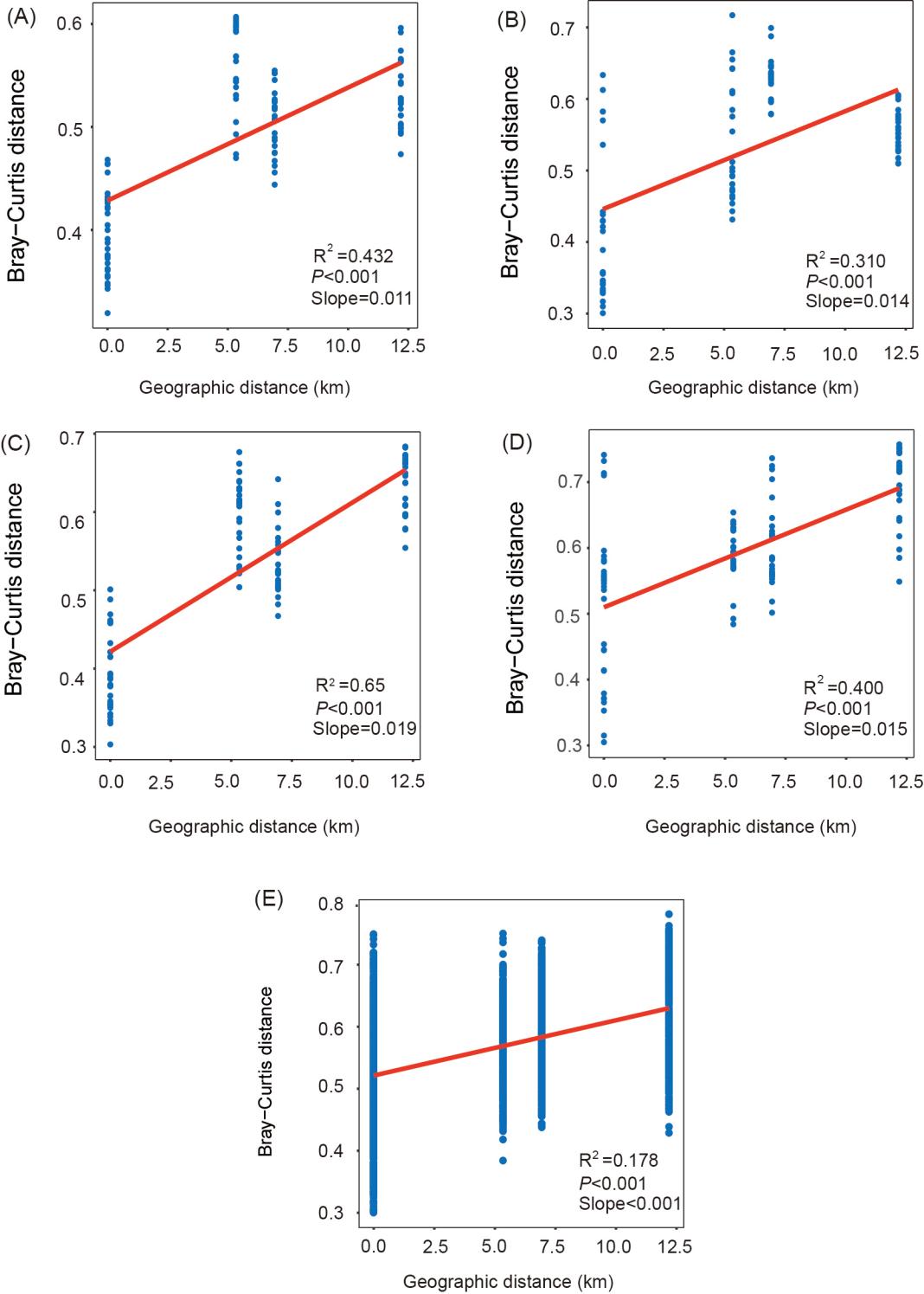


**Fig. S9** Correlation of Bray-Curtis distances with geographic distance. (A) *A. corniculatum*, (B) *B. sexangular*, (C) *K. obovata*, (D) bulk samples and (E) All.


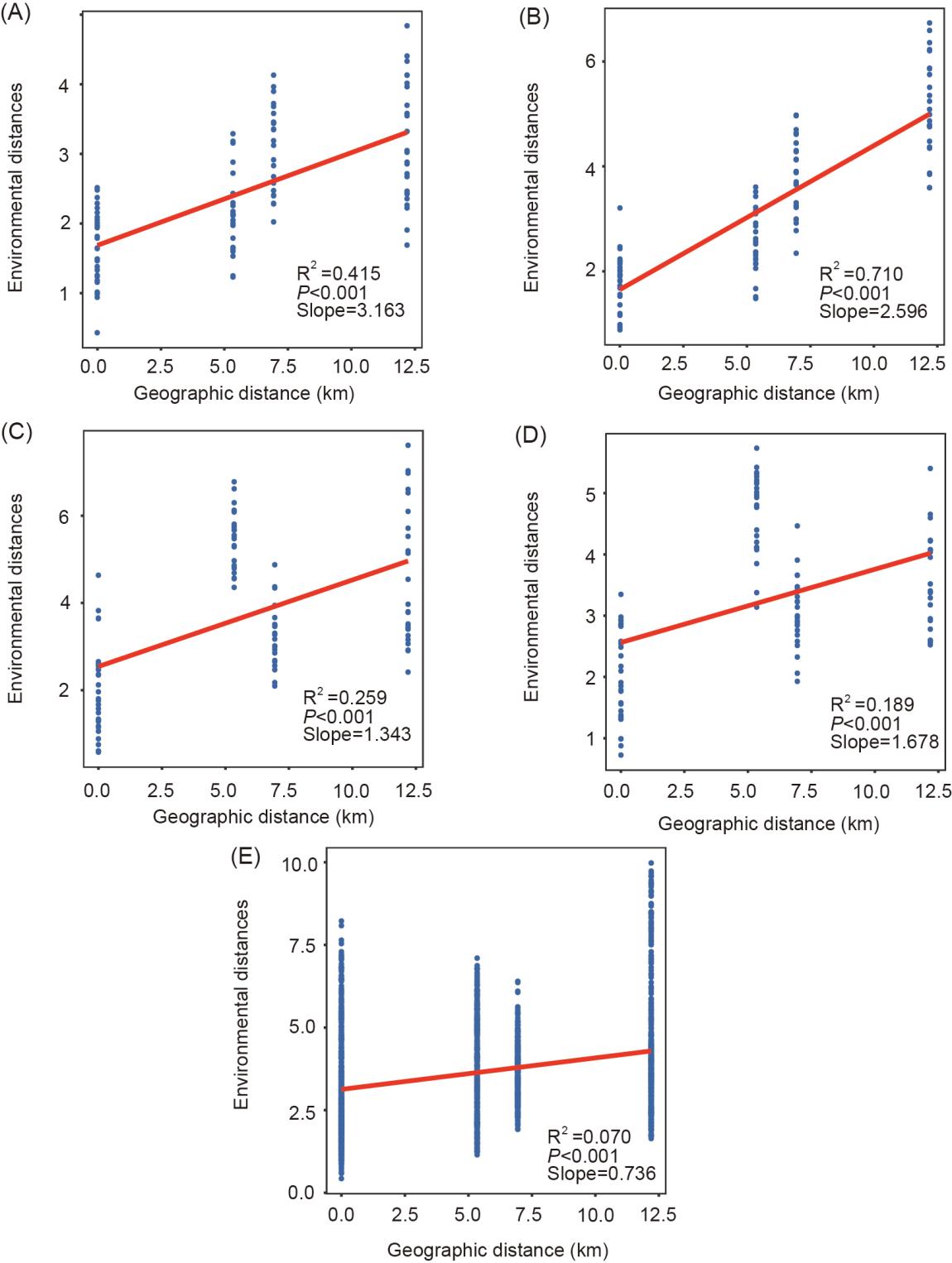


**Fig. S10** Correlation of environmental distances with geographic distance. (A) *A. corniculatum*, (B) *B. sexangular*, (C) *K. obovata*, (D) bulk samples and (E) All.


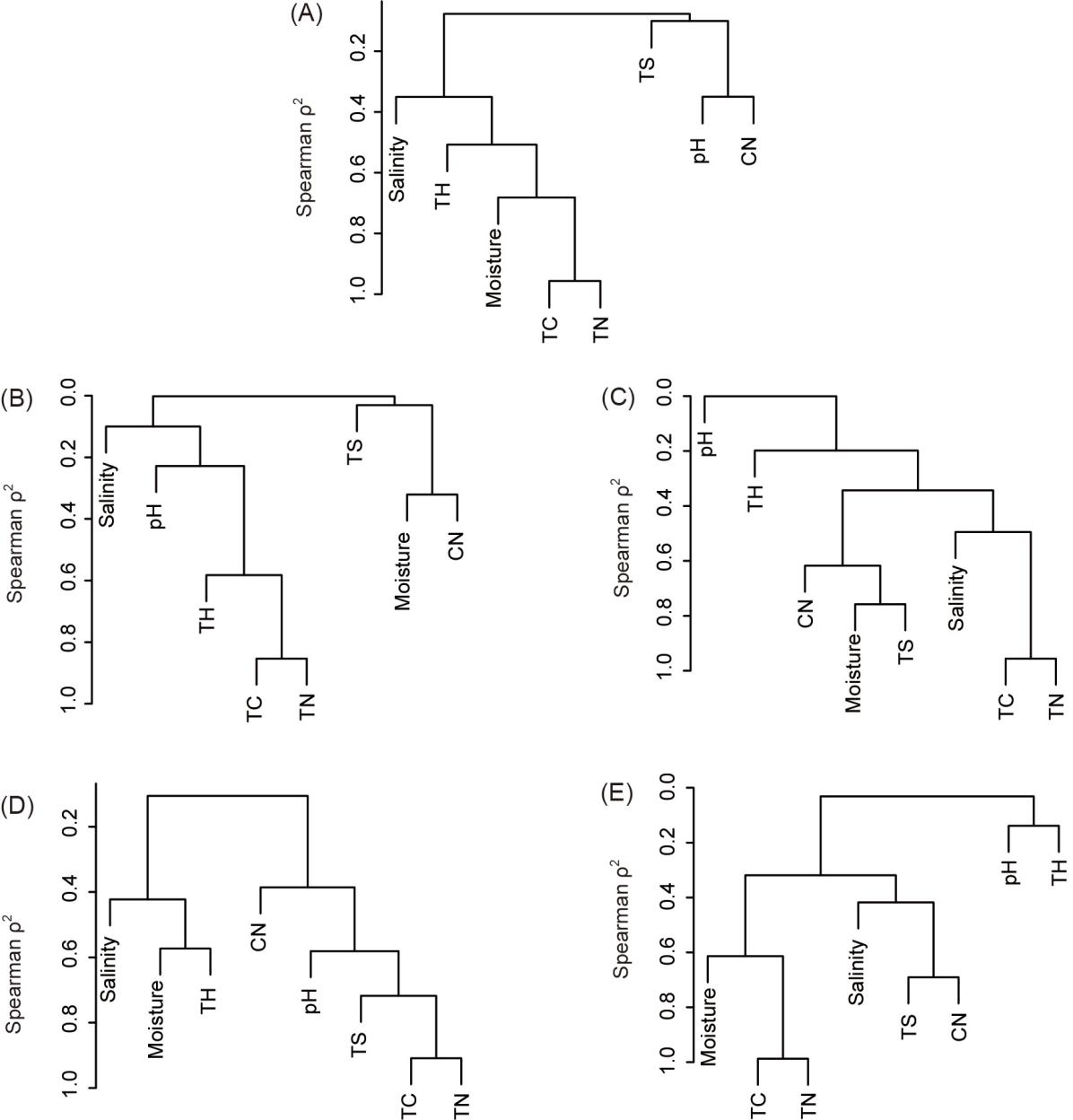


**Fig. S11** Clustering the environmental variables of all (A), *A. corniculatum* (B), *B. sexangular* (C), *K. obovata* (D) and bulk (E) sediments. TC, total carbon; TH, total hydrogen; TN, total nitrogen; TS, total sulfur; CN, carbon/nitrogen.


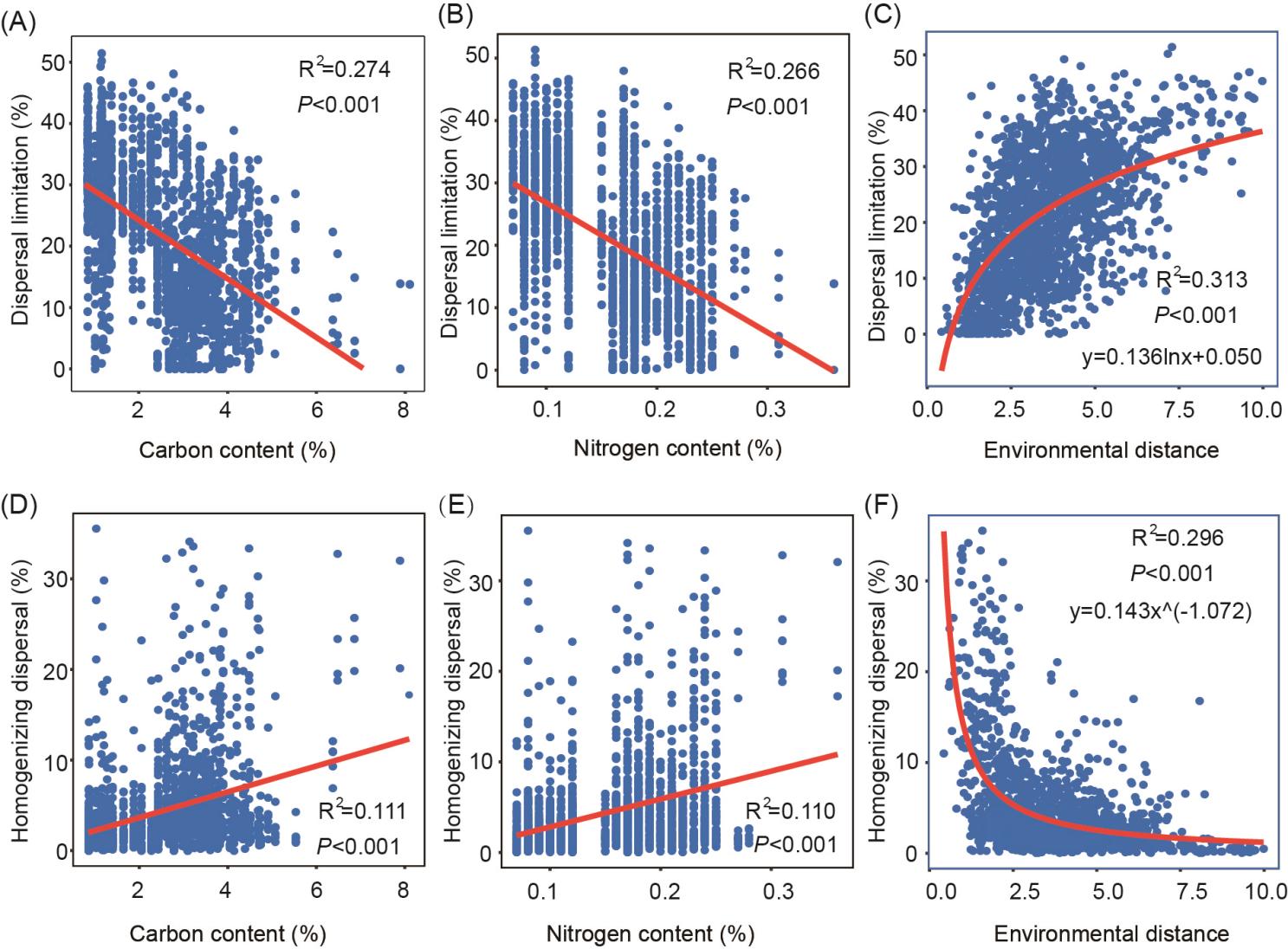


**Fig. S12** Correlation between bacterial dispersal ability and sediment physicochemical conditions. The correlation between the relative importance of dispersal limitation and homogenizing dispersal with (A,D) carbon, (B,E) nitrogen content and (C,F) environmental distance. For each pair of sediment samples, the lower value of carbon and nitrogen content of the two samples was used.


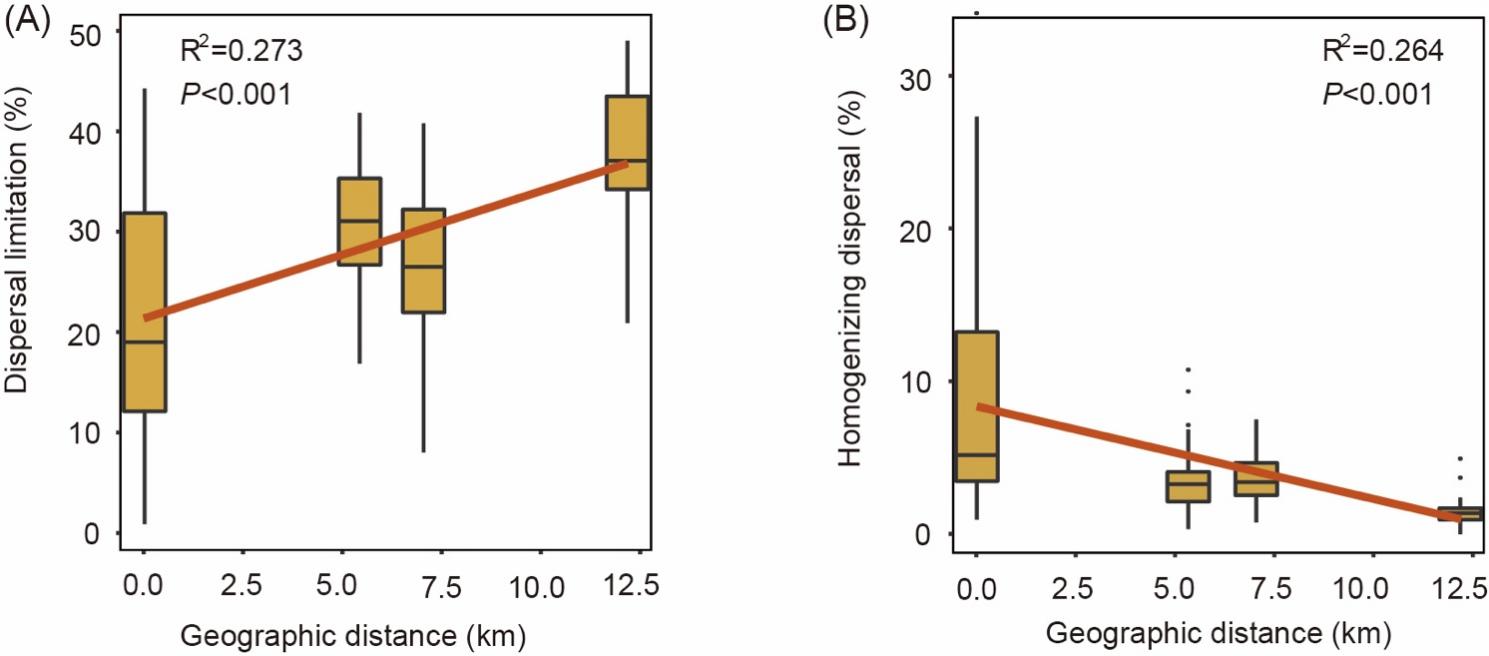


**Fig. S13** Correlation between relative importance of different assembly mechanisms in bulk samples. (A) dispersal limitation and (B) homogenizing dispersal.

**
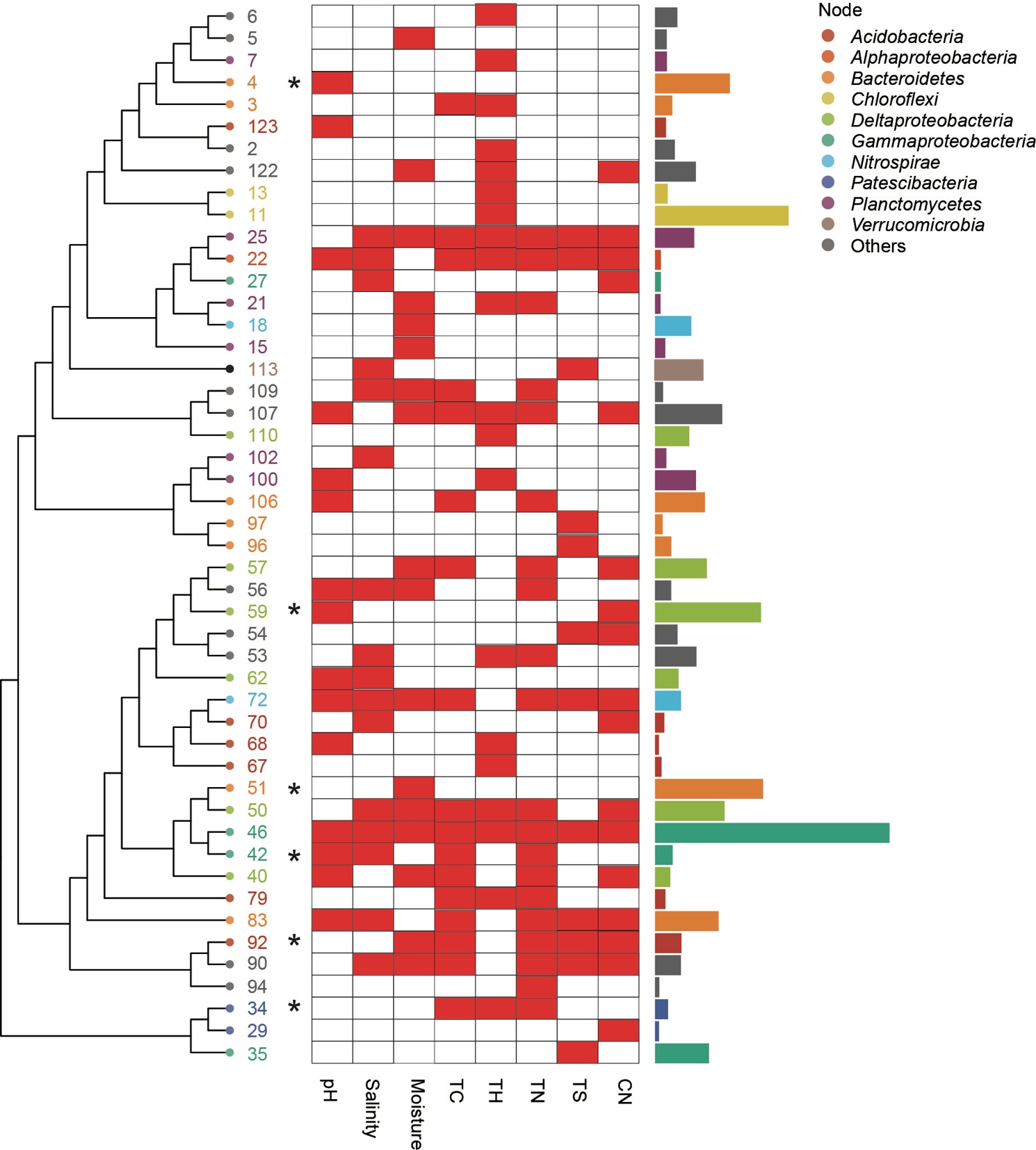
**

**Fig. S14** Environmental conditions essential to bacteria in different phylogenetic bins. A red cell indicates a significant correlation between the bin and the corresponding physicochemical condition. The bins with asterisks are dominated by the homogeneous selection, while the others are dominated by drift. The bars on the right indicate abundance.


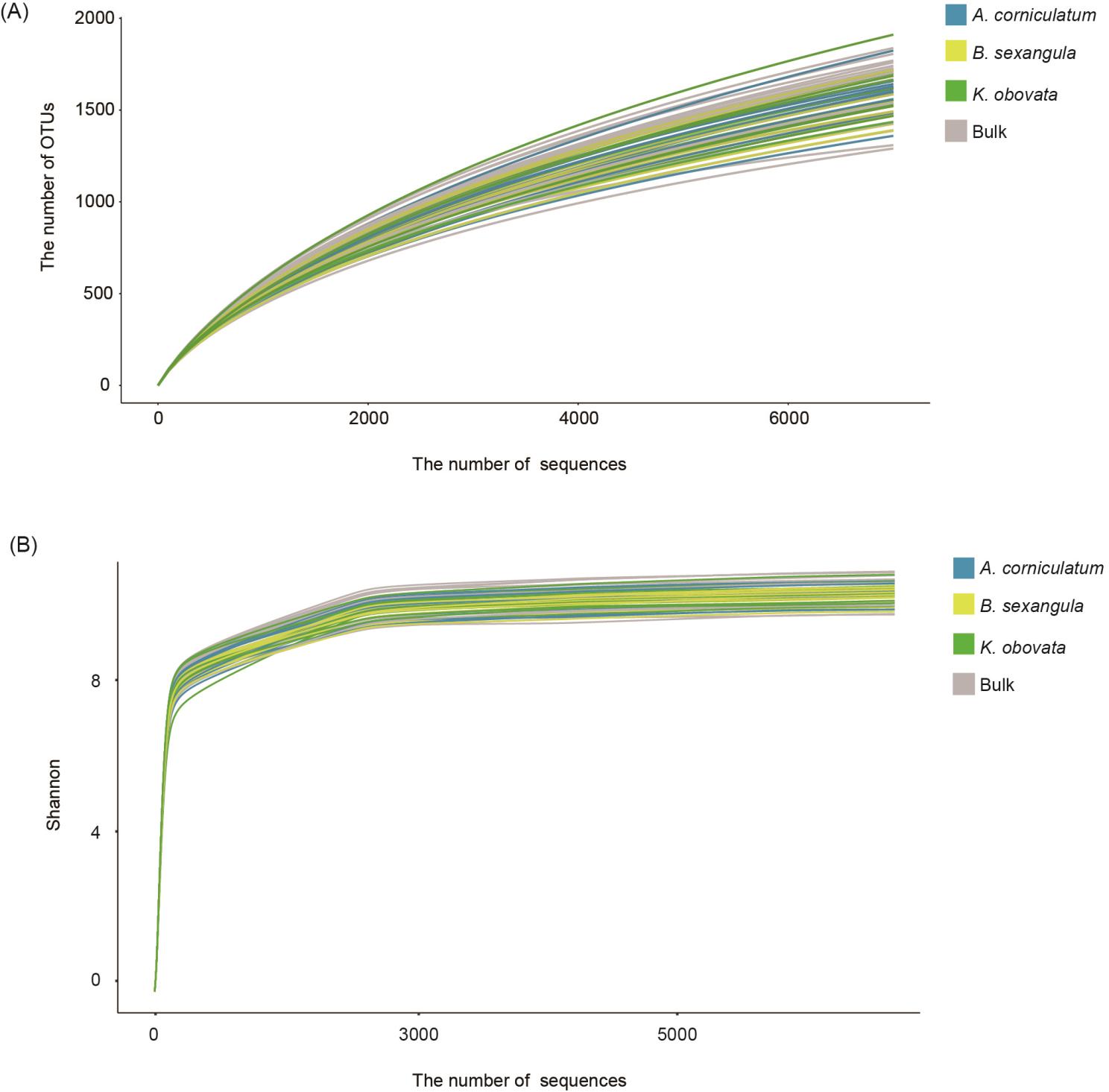


**Fig. S15** the rarefaction curves of the four sediment types.
